# Lagrangian and Eulerian dataset of the wake downstream of a smooth cylinder at a Reynolds number equal to 3900

**DOI:** 10.1016/j.dib.2021.107725

**Published:** 2021-12-16

**Authors:** Ali Rahimi Khojasteh, Sylvain Laizet, Dominique Heitz, Yin Yang

**Affiliations:** a INRAE, OPAALE, 17 avenue de Cucillé, Rennes 35044, France; b Turbulence Simulation Group, Department of Aeronautics, Imperial College London, Exhibition Road, London SW7 2AZ, United Kingdom

**Keywords:** Lagrangian particle trajectory, Direct Numerical Simulation, Synthetic particle transport, Turbulent wake flow

## Abstract

The dataset contains Eulerian velocity and pressure fields, and Lagrangian particle trajectories of the wake flow downstream of a smooth cylinder at a Reynolds number equal to 3900. An open source Direct Numerical Simulation (DNS) flow solver named Incompact3d was used to calculate the Eulerian field around the cylinder. The synthetic Lagrangian tracer particles were transported using a fourth-order Runge-Kutta scheme in time and trilinear interpolations in space. Trajectories of roughly 200,000 particles for two 3D sub-domains are available to the public. This dataset can be used as a test case for tracking algorithm assessment, exploring the Lagrangian physics, statistic analyses, machine learning, and data assimilation interests.

## Specifications Table

**Table:** 

Subject	Physics
	Engineering
Specific subject area	4D Particle Tracking Velocimetry (4D-PTV)
	Lagrangian Particle Tracking (LPT)
	Direct Numerical Simulation (DNS)
Type of data	Text file
How data were acquired	Direct Numerical Simulation (DNS)
	Synthetic particle transport
Data format	Raw
Parameters for data collection	The Eulerian velocity and pressure fields as well as the Lagrangian trajectories were collected for every 10 and 1 DNS time steps in sub-domain 1 and sub-domain 2, respectively.
Description of data collection	The Eulerian dataset was computed by an open-source Direct Numerical Simulation (DNS) code, named Incompact3d. The Eulerian velocity and pressure snapshots of two sub-domains were collected in the Data INRAE repository [1]. Nearly 200,000 synthetic Lagrangian trajectories were transported and saved for each sub-domain.
Data source location	Institution: French National Institute for Agriculture, Food, and Environment (INRAE)
	City/Town/Region: Rennes
	Country: France
Data accessibility	Repository name: Data INRAE
	Direct URL to data: https://doi.org/10.15454/GLNRHK
	Instructions for accessing these data: Free access
Related research article	Ali Rahimi Khojasteh, Yin Yang, Dominique Heitz, and Sylvain Laizet, “Lagrangian coherent track initialization”, Physics of Fluids 33, 095113 (2021) [2]https://doi.org/10.1063/5.0060644

## Value of the Data

• Recent rapid development in time-resolved three-dimensional Particle Tracking Velocimetry (4D-PTV) and Particle Image Velocimetry (PIV) studies arises a need to have ground truth datasets. To this end, a reference dataset was generated from a highly-resolved Direct Numerical Simulation (DNS).
• The data can be used by the PIV/PTV algorithm developers for assessment and validation purposes, as well as by those interested in machine learning and data assimilation studies in fluid mechanics. Moreover, scientists can benefit from the Eulerian and Lagrangian snapshots in the dataset to explore the physics of turbulent wake flows.
• Four types of data including, Lagrangian trajectories, 3D velocity fields, 2D velocity snapshots, and pressure fields, in two sub-domains are available in the repository. As listed in Table 1, one or more types of data can be used depending on the application.

**Table 1:** Application of the current dataset in PIV / PTV community.

	Lagrangian	3D velocity	2D velocity	Pressure	Target
Dataset application	trajectory	field	snapshot	field	studies
4D-PTV algorithm assessment	✓	✗	✗	✗	[2], [3], [4], [5]
Volumetric pressure from PTV	✓	✗	✗	✓	[6], [7]
4D flow field reconstruction	✓	✓	✗	✗	[8]
Lagrangian physics	✓	✗	✗	✗	[9], [10]
Machine learning	✓	✗	✓	✗	[11], [12]
Eulerian physics	✗	✓	✓	✗	-
Data assimilation	✓	✓	✗	✓	[13]
CFD assessment	✗	✓	✗	✓	-
2D2C-2D3C-PIV	✓	✓	✓	✗	[14]
Tomo-PIV	✓	✓	✗	✗	[14], [15]
• There is an open-access Lagrangian particle transport software package in the data repository if interested readers require tracer particle trajectories with different properties including particle concentrations, temporal scale, and noise level.

## 1 Data Description

**Fig. 1:**
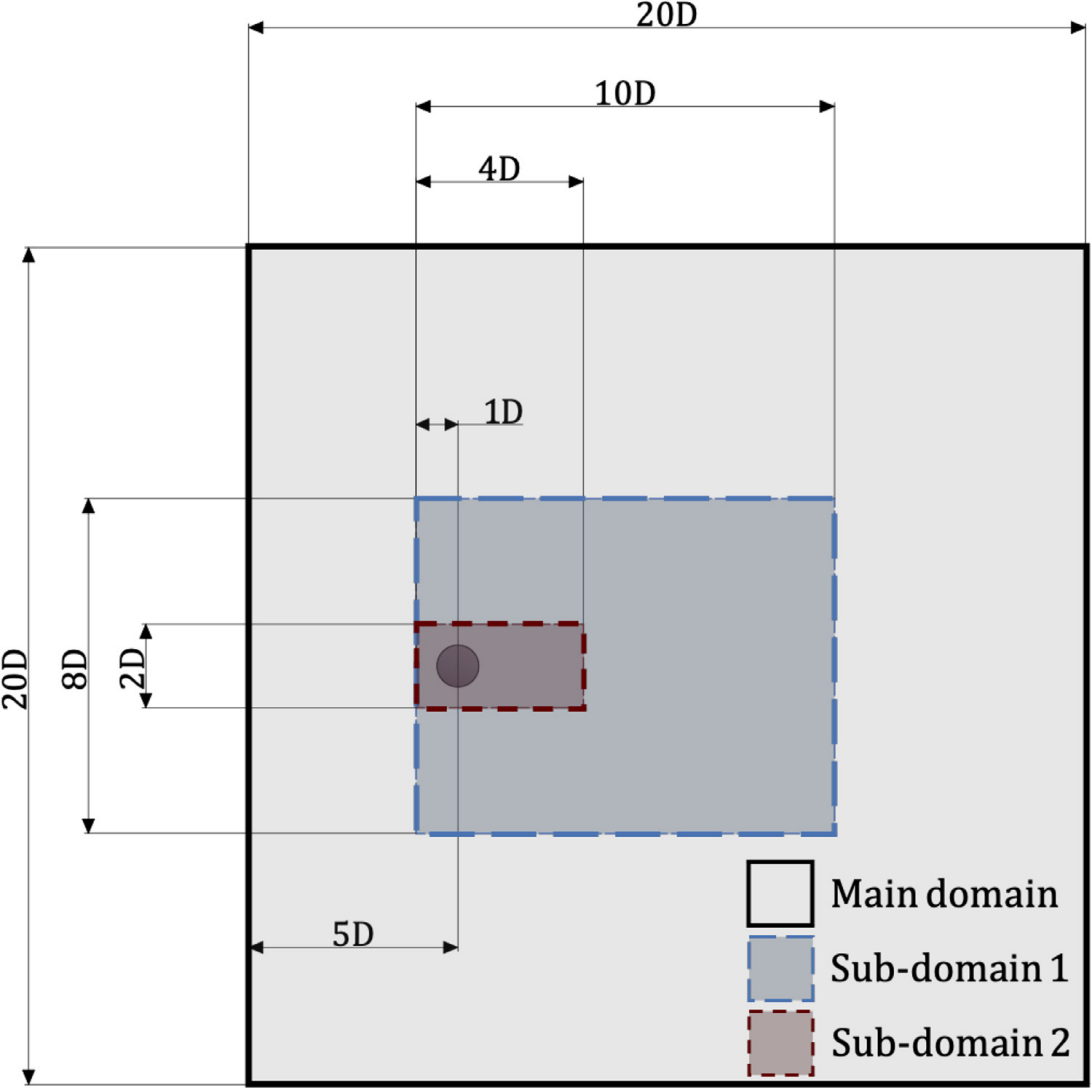
Dimensions of two computational sub-domains from the flow over a smooth cylinder at subcritical Reynolds number 3900.

**Fig. 2:**
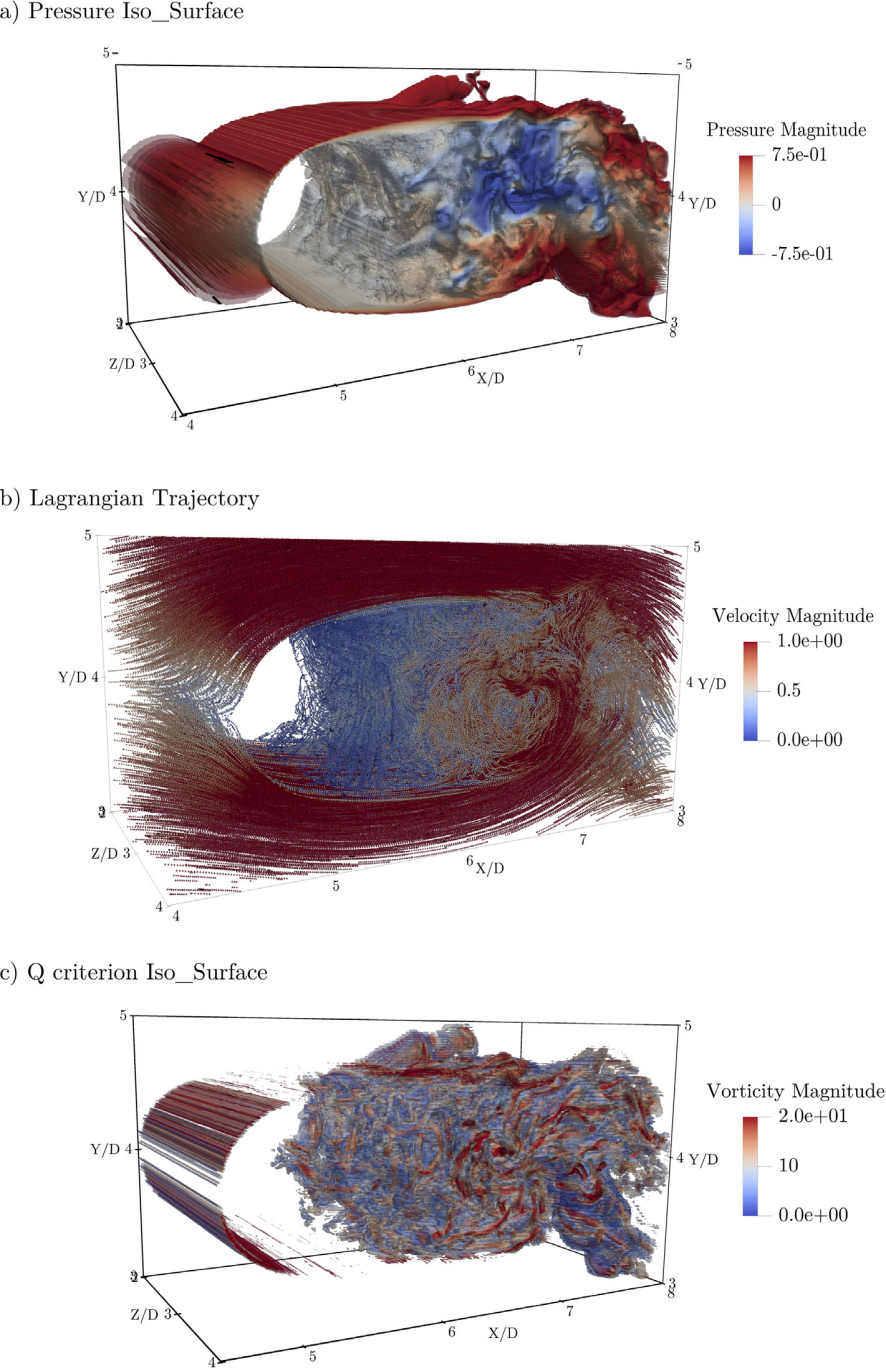
Snapshot view of Sub-domain 2: (a), Pressure iso-surface coloured by the magnitude of pressure; (b), Lagrangian trajectories of 20,000 particles after 1000 DNS time steps coloured by the velocity magnitude; (c), Q criterion representation of the Eulerian flow structures coloured by the vorticity magnitude.

**Fig. 3:**
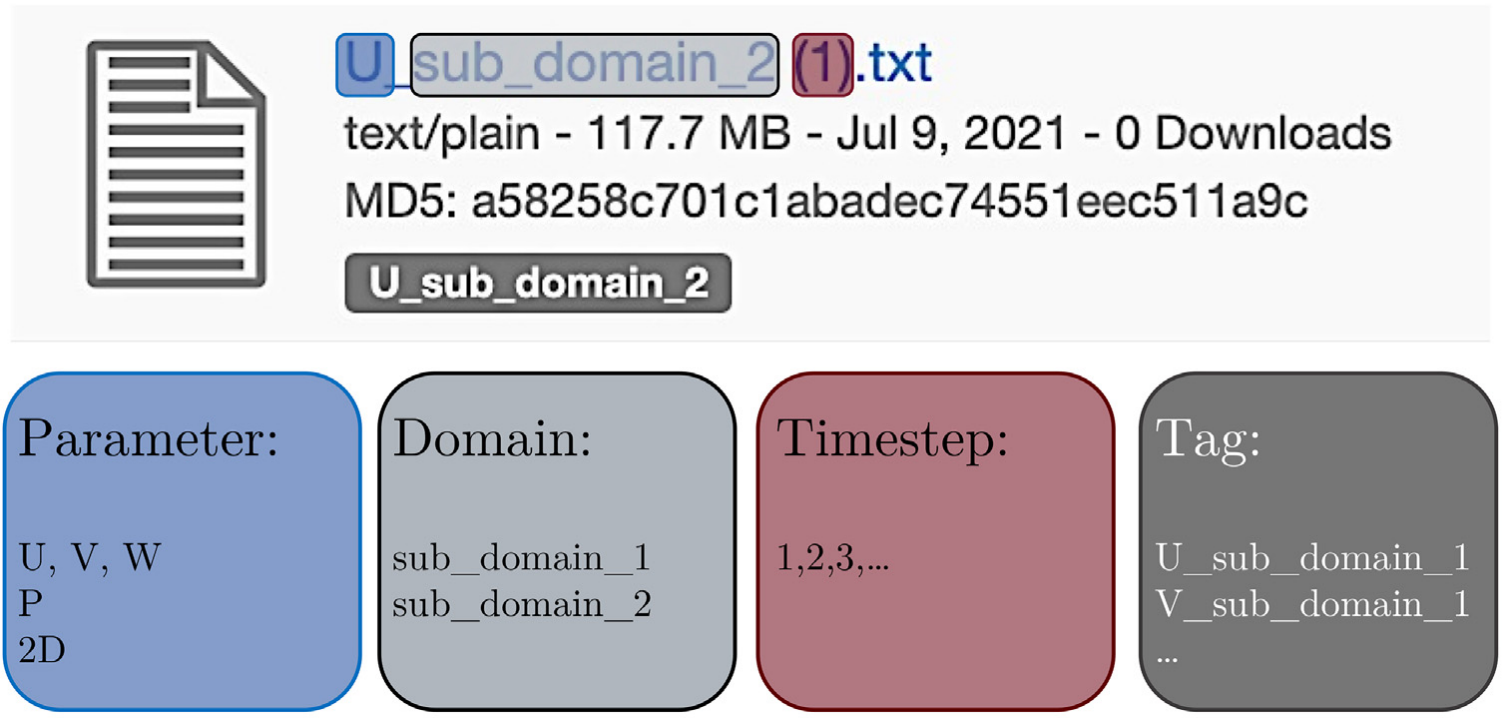
The naming format of each snapshot in the data repository.

A highly-resolved Direct Numerical Simulation (DNS) of the flow over a smooth cylinder at a subcritical Reynolds number 3900 (based on the diameter D of the cylinder and the free-stream velocity) was performed to generate the data. Double-precision Eulerian and Lagrangian fields for two sub-domains were collected, as shown in Fig. 1. The dimensions of Sub-domain 1 are 10D×8D×6D. Data were saved every 10 DNS time steps for Sub-domain 1 due to online cloud storage limitation (saving every time step would have required roughly 30 Tb of storage per vortex shedding). 1000 snapshots were also collected for a smaller sub-domain with dimensions of 4D×2D×2D (i.e., Sub-domain 2) for every DNS time step. Sub-domain 2 is suitable for studies requiring the highest possible temporal resolution. Details of two sub-domains can be found in Table 2. One Eulerian snapshot of the current wake flow is shown in Fig. 2. For both sub-domains, Lagrangian trajectories are provided for roughly 200,000 synthetic particles. Three main categories are available in in the data repository, Sub-domain-1, Sub-domain-2, and Software. The snapshots are formatted in text (.txt) and collected in compressed files (.zip). There is no particular requirement for reading and opening the data. The naming format of each snapshot is shown in Fig. 3. The Eulerian 3D snapshots are saved in vector formats. Therefore, it is necessary to extract them within three internal loops in xyz directions. The users also need to download the grid file separately to find the corresponding coordinates.

**Table 2:** Domain specification.

Domain	Dimension			Grid size			Time step	Eulerian snapshot size
	x	y	z	nx	ny	nz	dt	
Computation Domain	20D	20D	6D	1537	1025	256	$0.00075\;D/U_{\infty }$	12.9 Gb
Sub-domain 1	(4-14)D	(6-14)D	6D	769	777	256	$0.0075\;D/U_{\infty }$	4.8 Gb
Sub-domain 2	(4-8)D	(9-11)D	(2-4)D	308	328	87	$0.00075\;D/U_{\infty }$	256 Mb

## 2 Experimental Design, Materials and Methods

The PIV/PTV community consistently requires synthetic datasets to assess and validate developed image based methods. The EUROPIV Synthetic Image generator (SIG) developed a standardised synthetic dataset framework for the PIV/PTV community [16]. SIG targeted three objectives including, algorithm performance assessment, algorithm sensitivity analysis as a function of characteristic parameters, and algorithm comparison. Characteristic parameters refer to particle concentration (i.e., density), temporal scale, and noise ratio that can determine how the synthetic dataset is similar to a real experiment. Since then, by increasing capabilities of the PIV/PTV techniques, algorithm assessments constantly require datasets of flows with relatively complex and high gradient regions associated with 3D directional dynamics. That was the motivation to generate a database of Eulerian velocity and pressure fields with Lagrangian trajectories for the wake carrying complex flow motions downstream of a smooth cylinder. Applications of the current dataset can be summarised in Table 1.

### 2.1 Eulerian method

The computations are carried out with the open-source flow solver named Incompact3d [17], [18] based on sixth-order finite-difference compact schemes for the spatial discretisation on a Cartesian grid. Simplicity of the Cartesian grid offers the ability of implementing higher order spectral schemes for spatial discretisation. For the current simulation, the time advancement was performed with an explicit third-order Adams Bashforth scheme. The governing equations are solved with a fractional step method to treat the incompressibility constraint, which requires solving an additional projection step, the Poisson equation. This Poisson equation is fully solved in spectral space using three-dimensional Fast Fourier Transforms (FFTs). In the present work, the smooth cylinder is modelled using a customised immersed boundary method (IBM) with an artificial flow inside the cylinder to ensure the smoothness of the velocity field while imposing a no-slip boundary condition at the cylinder. More details about the flow solver can be found in Laizet and Lamballais [17]. Incompact3d is built with a powerful 2D domain decomposition for simulations on super-computers. The computational domain is split into a number of sub-regions (pencils) which are each assigned to an MPI process. The derivatives and interpolations in the x-direction (y-direction, z-direction) are performed in X-pencils (Y-pencils, Z-pencils), respectively. The 3D FFTs required by the Poisson solver are also broken down as series of 1D FFTs computed in one direction at a time. Global transpositions to switch from one pencil to another are performed with the MPI command MPI_ALLTOALL(V). Incompact3d can scale well with up to hundreds of thousands of MPI processes for simulations with several billion grid nodes [18]. Inflow/outflow boundary conditions are implemented along the streamwise direction with free-slip and periodic boundary conditions along the vertical and spanwise directions, respectively. The simulation was performed on nearly $4\times 10^{10}$ grid points (see Table 2). The grid was uniform in the streamwise and spanwise directions, while a non-uniform grid was used in the vertical direction, with a grid refinement towards the centre of the cylinder. The finest grid size in the vertical direction was $\Delta y_{\min}=0.00563D$. The dimensional DNS time step was $0.00075D/U_{\infty }$ (where $U_{\infty }$ is the free-stream velocity). It takes 6667 DNS time steps to simulate one vortex shedding. It should also be mentioned that 1333 DNS time steps correspond to one integral temporal scale $D/U_{\infty }$.

### 2.2 Lagrangian method

#### 2.2.1 Particle transport

The Johns Hopkins Turbulence Database (JHTDB) generated from DNS has been employed widely for the quantitative performance assessment of PIV/PTV algorithms [19]. JHTDB contains nine multi-terabyte datasets in turbulent cases such as homogeneous isotropic turbulence (HIT) and channel flows. The current study brings added value to the available databases [16], [19], [20] by providing a case in the wake flow. Numerous complexities occur in the wake behind the cylinder at subcritical Reynolds number, which can be a challenging test case for quantitative assessments.

In the present dataset, synthetic particles were transported using a conventional fourth-order Runge-Kutta scheme in time. The Lagrangian velocities of the synthetic particles were calculated by trilinear spatial interpolations over eight nearest neighbour grid points. To mimic the real experimental condition, three characteristic parameters, temporal scale, particle concentration, and noise ratio must be defined. Depending on the desired temporal scale, the synthetic time step can be calculated by knowing that the DNS time step of the current dataset is roughly 20 times smaller than the Kolmogorov temporal scale. The desired temporal scale should be defined based on experimental hardware facilities, such as the illumination pulse rate or the camera frequency. The particle concentration also can be computed based on the number of particles per Kolmogorov length scale ($\text{pp}\eta^{3}$). The Kolmogorov length scale is almost 2.8 times smaller than the average grid size in the vertical direction for the current dataset. In a real experiment, the achievable spatial resolution is highly limited by the particle seeding system and the PIV/PTV algorithm performance. Therefore, an appropriate number of synthetic particles in the domain can be selected depending on the desired spatial resolution. An open-access tracer particle transport software package in MATLAB graphical user interface (GUI) is available as an additional tool in the data repository. Interested users can create tracer particle trajectories with different properties including particle concentrations up to the DNS spatial resolution, temporal scale up to the DNS time scale, and noise level.

#### 2.2.2 Particle transport accuracy

**Fig. 4:**
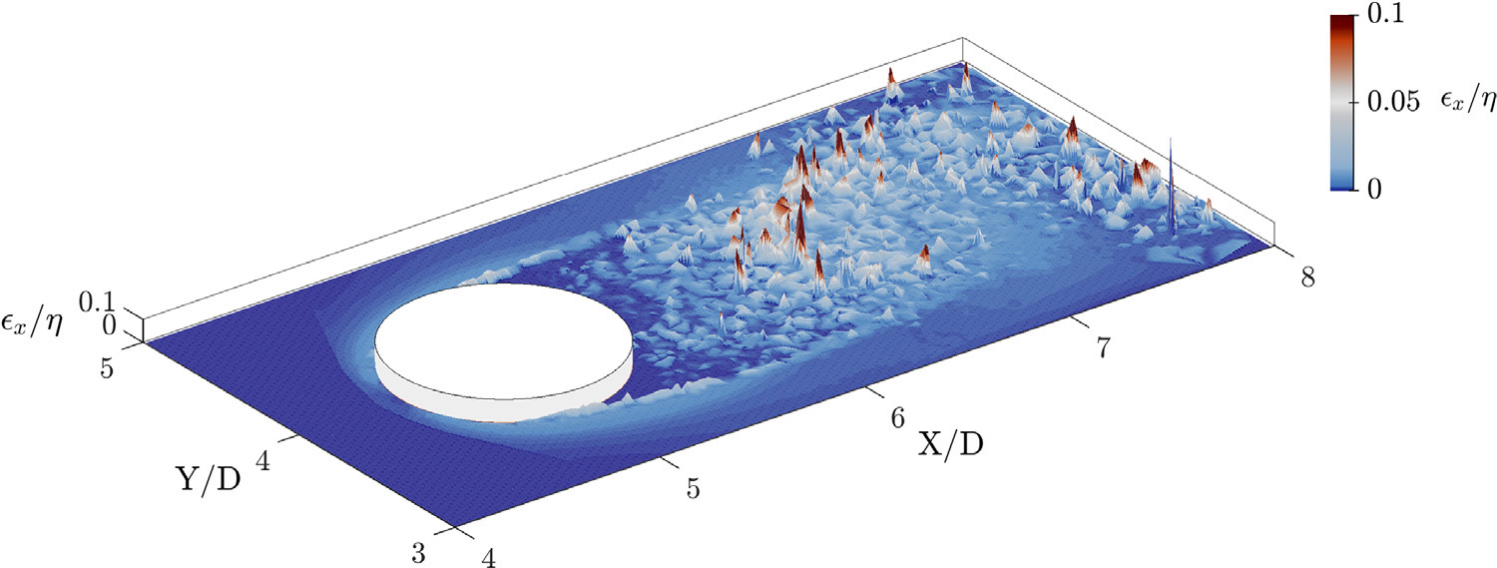
2D map of the non dimensional position error of Lagrangian transport computed every 10 DNS time step after 1000 time steps and after an average in the spanwise direction.

A comparison was made between the transport of particles at every 10 DNS time step (i.e., temporal scale of Sub-domain 1) with the transport of particles at every DNS time step in Sub-domain 2, to quantify the uncertainty level of trajectories in Sub-domain 1. As a result, the mean deviation of the trajectories between two temporal scales after 1000 DNS time steps in the larger domain is equal to 3.28η, with η the Kolmogorov spatial scale. The standard deviation of position error is $\sigma_{\epsilon }=0.017\eta$. Fig. 4 shows a 2D map of the non-dimensional position deviation ϵ/η between two temporal scales averaged in the spanwise direction. Therefore, it is recommended to use the data from Sub-domain 2 for studies requiring accurate trajectories inside the wake region, while the data from Sub-domain 1 are better suited for studies focusing on large scale motions.

## Ethics Statement

This study did not conduct experiments involving humans and animals.

## CRediT authorship contribution statement

**Ali Rahimi Khojasteh:** Conceptualization, Methodology, Investigation, Visualization, Writing – original draft. **Sylvain Laizet:** Methodology, Investigation, Writing – review & editing. **Dominique Heitz:** Methodology, Supervision, Writing – review & editing. **Yin Yang:** Methodology, Writing – review & editing.

## Declaration of Competing Interest

The authors declare that they have no known competing financial interests or personal relationships that could have appeared to influence the work reported in this paper.

## Appendix A Supplementary materials

Supplementary Data S1 Supplementary Raw Research Data. This is open data under the CC BY license http://creativecommons.org/licenses/by/4.0/ Supplementary Data S1
