# Supplementary material for: Lagrangian and Eulerian dataset of the wake downstream of a smooth cylinder at a Reynolds number equal to 3900
Source: Data Brief. 2021 Dec 16;40:107725. doi: 10.1016/j.dib.2021.107725 (PMC8713130; doi:10.1016/j.dib.2021.107725)
Supplement: Supplementary Data S1 — Supplementary Raw Research Data. This is open data under the CC BY license http://creativecommons.org/licenses/by/4.0/ [file mmc1.pdf]

Physics of Fluids

Lagrangian coherent track initialization

Cite as: Phys. Fluids **33**, 095113 (2021); https://doi.org/10.1063/5.0060644
Submitted: 21 June 2021 . Accepted: 21 August 2021 . Published Online: 09 September 2021

Ali Rahimi Khojasteh, Yin Yang, Dominique Heitz, Sylvain Laizet, et al.

COLLECTIONS

F This paper was selected as Featured

View Online Export Citation CrossMark

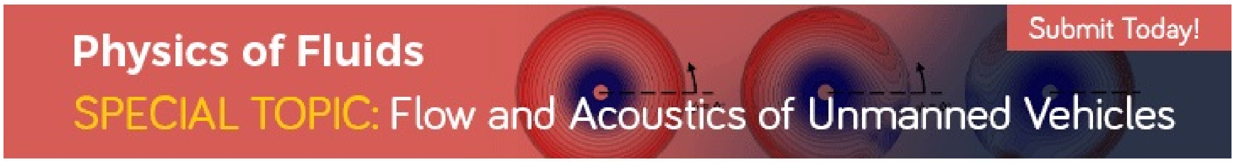

AIP Publishing

© 2021 Author(s).

Lagrangian coherent track initialization

Cite as: Phys. Fluids **33**, 095113 (2021); doi: 10.1063/5.0060644
Submitted: 21 June 2021 · Accepted: 21 August 2021 ·
Published Online: 9 September 2021

View Online Export Citation CrossMark

Ali Rahimi Khojasteh,[1,a)] Yin Yang,[1] Dominique Heitz,[1] and Sylvain Laizet[2]

AFFILIATIONS

[1]INRAE, OPAALE, 17 avenue de Cucillé, 35044 Rennes, France
[2]Turbulence Simulation Group, Department of Aeronautics, Imperial College London, Exhibition Road, London SW7 2AZ, United Kingdom

[a)]Author to whom correspondence should be addressed: ali.rahimi-khojasteh@inrae.fr

ABSTRACT

Advances in time-resolved three-dimensional Particle Tracking Velocimetry (4D-PTV) techniques have consistently revealed more accurate Lagrangian particle motions. A novel track initialization technique as a complementary part of 4D-PTV, based on local temporal and spatial coherency of neighbor trajectories, is proposed. The proposed Lagrangian Coherent Track Initialization (LCTI) applies physics-based Finite Time Lyapunov Exponent (FTLE) to build four frame coherent tracks. We locally determine Lagrangian coherent structures among neighbor trajectories by using the FTLE boundaries (i.e., ridges) to distinguish the clusters of coherent motions. To evaluate the proposed technique, we created an open-access synthetic Lagrangian and Eulerian dataset of the wake downstream of a smooth cylinder at a Reynolds number equal to 3900 obtained from three-dimensional direct numerical simulation. Performance of the proposed method based on three characteristic parameters, temporal scale, particle concentration (i.e., density), and noise ratio, showed robust behavior in finding true tracks compared to the recent initialization algorithms. Sensitivity of LCTI to the number of untracked and wrong tracks is also discussed. We address the capability of using the proposed method as a function of a 4D-PTV scheme in the Lagrangian particle tracking challenge. We showed that LCTI prevents 4D-PTV divergence in flows with high particle concentrations. Finally, the LCTI behavior was demonstrated in a jet impingement experiment. LCTI was found to be a reliable tracking tool in complex flow motions, with a strength revealed for flows with high velocity and acceleration gradients.

Published under an exclusive license by AIP Publishing. https://doi.org/10.1063/5.0060644

I. INTRODUCTION

Recent developments in the Shake-The-Box (STB) approach[1] have led to renewed attention to time-resolved three-dimensional Particle Tracking Velocimetry (4D-PTV) for the study of turbulent flows. STB introduces a fast and efficient tracking idea based on a particle position prediction step followed by an image space optimization scheme solved with "Shaking." STB was first initiated and shaped after the Iterative Particle Reconstruction (IPR) concept proposed by Wieneke.[2] 4D-PTV methods like STB and Kernelized Lagrangian Particle Tracking (KLPT, Yang and Heitz[3]) require an appropriate and reliable number of initialized true tracks at every time step. Otherwise, the tracking process fails to reconstruct trajectories for the majority of particles.[1] Such a failure illustrates the importance of implementing a robust multi-frame track initialization technique to prevent 4D-PTV divergence, particularly in dense and complex situations.

The idea of initializing a possible track in four frames, known as four frame best estimate (4BE), has been widely used in Lagrangian Particle Tracking (LPT)/PTV studies.[4–8] Four frame tracking methods with simple nearest neighbor particle matching have been introduced for low density and smooth flow behaviors.[4] However, the nearest neighbor fails to reconstruct true trajectories in high particle concentrations if the length between neighbor particles has the same order of their trajectory displacement within two time steps. New studies recently improved four frame track initialization performance, including four-frame Best Estimate Nearest Neighbor Initialization[5] (4BE-NNI method) by looking for the nearest neighbors in sequential frames until a unique solution is found or Enhanced Track Initialization[7] (4BE-ETI method) by looking for all track possibilities with an adjustable search volume (see also Ref. 8). Dou *et al.*[6] proposed initializing with two nearest candidates in a similar spirit and then kept predicting and particle matching in the next two following time steps (four frames in total) until a unique match is found. Cierpka *et al.*[9] have shown that the four frame methodology could be extended to multi-frame tracking with the combination of neighbor possibility and temporal prediction in sequential steps. The most straightforward prediction function is the linear predictor that can be calculated from the position difference of every two possible matches. The use of a linear predictor improved the probability of finding true tracks as well as reducing the computation time by

having a targeted search volume.[9] Some studies also suggest applying the prior Particle Image Velocimetry (PIV) velocity field as a predictor.[1,10] Although this idea is applicable to two-dimensional (2D) and three-dimensional (3D) studies, extracting a 3D PIV velocity field is expensive due to its spatial resolution, uncertainty, and complex experimental issues. For complex flow motions and experiments with high particle densities, a further step for the track validity check is required to avoid false detection. Guezennec *et al.*[11] originated this validity check as a self-coherency algorithm with the concept of path coherence. Their technique minimizes a penalty function from all possibilities in five frames. In their study, a possible track is a coherent path if it is a smooth trajectory in position, velocity, acceleration, and rate of acceleration. This spatial and temporal self-consistency penalty function only focuses on a single track behavior. Recent four frame based techniques also performed similar self-consistency approaches. As an example, Dou *et al.*[6] checked if the velocity differences between two frames exceed a certain threshold to validate a possible track. With a comparative approach, Ouellette *et al.*[5] controlled the acceleration change instead of velocity. Likewise, the self-consistency approach is used in two frame based tracking techniques.[12] These treatments rely on spatial and temporal filtering of the trajectories to avoid false tracks. However, as complex flows often feature high gradient motions, over-smoothing dynamics could lead to a quality degeneration of the reconstructed field. The degeneration problem is that there is no direct learning based on the physics of flow included in the classical four frame schemes, which brings more challenges for complex and high-density flow motions.

We argue that when a track cannot be reconstructed successfully solely due to ambiguities caused by overlapping and multi-possibilities, it is always beneficial to extract more information from its neighborhood. If the reconstructed tracks are available in the neighborhood, we can use this information to gain a better insight into the target particle's potential behavior and eventually solve the ambiguity problematic associated with the initialization. As we are dealing with the fluid flow, assuming a constant solid local rigid neighborhood is naive and erroneous, it is essential to consider a coherent neighborhood where both the target particle and neighbor particles share the coherent motion.

This paper seeks to increase the track initialization accuracy and robustness in flows with high particle concentration, large temporal scale, and noisy reconstruction featured by complex motions, such as high velocity and acceleration gradients. Therefore, we propose a novel Lagrangian Coherent Track Initialization (LCTI) technique for finding tracks in four (or multi) frames that belong to clusters of coherent motions. We apply Lagrangian Coherent Structures[13] (LCS) to distinguish coherent and non-coherent neighbor trajectories. The LCS, also known as the skeleton of flow, determines separatrix lines or surfaces that divide flow structures into different coherent regions. We use Finite Time Lyapunov Exponent (FTLE) that is the most common method in quantifying these separatrices' boundaries.[14] The paper is organized as follows: In Sec. II, we describe the methodology of the present study. In Sec. III, we discuss how to create synthetic datasets in the wake behind a smooth cylinder at a Reynolds number equal to 3900 (based on the cylinder diameter and freestream velocity). LCTI robustness, accuracy, and sensitivity analyses are also illustrated in terms of characteristic parameters. Finally, we demonstrate the LCTI performance for the LPT challenge wall-bounded wake flow behind a cylinder[15] and a jet impingement experiment in Secs. III and IV, respectively.

II. METHODOLOGY

In this section, we first address the 4D-PTV working principle and how the initialization step is employed in this process. Then we present the LCTI algorithm based on Lagrangian coherency in four frames in Sec. II B. We discuss in Sec. II C the concept of clusters of coherent particles and how to quantify the coherent trajectories.

A. 4D-PTV working principle

A typical 4D-PTV technique comprises four recursive steps inspired by STB, as shown in Fig. 1. These steps are particle reconstruction, track initialization, prediction, and optimization. Particle reconstruction with triangulation is a process that converts multi-view two-dimensional (2D) particle images to particle positions in a 3D domain. However, the triangulation only works for sparse particle concentrations lower than 0.001 particles per pixel (ppp). The number of ghost particles drastically increases in triangulation for higher particle concentrations due to overlapping particles. Hence, Wieneke[2] proposed IPR with an additional step to overcome the triangulation inaccuracy. In IPR, the triangulation is followed by an iterative optimization procedure that searches for the best particle position minimizing the intensity discrepancy between the original and the reprojected particle image. The reconstructed 3D positions from IPR are thereafter fed into the initialization part (see Fig. 1). After the tracklets of the first few frames are built, the prediction function then estimates positions of the next time step (t_{n+1}) using polynomial or Wiener filter predictors.[1,16] The optimization takes part from the predicted positions until the optimal positions at t_{n+1} are found. During the prediction-optimization phase, particles continuously enter the domain. Those new entry trajectories must be fed into the tracked poll; otherwise, there would eventually be no tracks left since all tracked particles would have left the domain for a flow with a main advection. In complex flow motions or with high particle concentrations, some particles lose their trajectories due to the optimization failure. In this scenario, the lost particles are kept in the residual images, but their tracks will be removed from the tracked poll (see Fig. 1). It is vital to reconstruct those lost particles and build tracklets since an increasing number of lost tracks will lead to the divergence of the tracking algorithm. To this

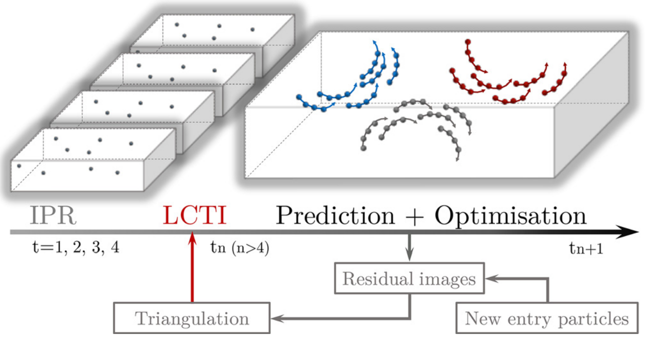

FIG. 1. 4D-PTV flow chart starting from particle reconstructions in four frames using IPR Wieneke.[2] LCTI recursively add new entry and lost tracks into the optimization poll.

Published under an exclusive license by AIP Publishing

end, the track initialization attempts to build tracklets for three types of particles, particles in the first four/five frames, new entries, and lost particles.

B. Track initialization methodology

The current initialization technique tries to find coherent tracklets in four frames. It should be noted that particles of a cluster are coherent if they spatially behave together over a finite time. A starting step is required in LCTI for the first time step t_1, where there is no neighbor track information. It can be done by a classic four-frame scheme with a narrow threshold to index the most reliable tracks. It is assumed that a track is relatively reliable if it has comparable small velocity and acceleration standard deviations in four time steps to avoid false tracks. The standard deviation of the particle image intensity can also determine whether a possible track is reliable. In practice, the LCTI steps can be listed as the following algorithm:

ALGORITHM 1: LCTI.

1: Index possible candidates inside the search volume at t_2;
2: Two consecutive predictions and candidate matching for t_3 and t_4;
3: Index possible tracks from t_1 to t_4;
4: Coherency check with neighbor tracks for each possible track;
5: Index the most coherent track.

Referring to the LCTI Algorithm 1, we need to define the search volumes to index possible candidates at each time step. Clark *et al.*[7] enhanced the probability of finding true tracks by applying adjustable anisotropic search volumes as a function of mean flow direction. Anisotropic means that if the mean flow (obtained from the predicted velocity) is dominant in one direction, the search volume in that direction is larger than in the other directions. Adjustable search volumes introduce local spatial motions (i.e., physics-based information) into four frame schemes, which can significantly tackle the high gradient threshold issues. On the other hand, using the adjustable search volume limits the number of possible candidates, avoiding non-coherent solutions by following the local spatial motion. The search volume in LCTI is based on the local maximum displacement map calculated from neighbor particles. Therefore, the first search volume is computed as a function of neighbor maximum displacements in each spatial direction between t_1 and t_2 as shown in Fig. 2(a). Then, every neighbor particle inside the search volume at t_2 is a candidate. These candidates are in one of the following categories: the true position of the target particle at t_2, the true position of other undetected tracks, and noise (i.e., false particle). Afterwards, a linear predictor [blue dash line arrows in Fig. 2(a)] between the target particle at t_1 and the possible candidate at t_2 is performed for every possible match. Similarly, the second search volume around the predicted position determines which particles are more likely to be in the true position at t_3. A possible track is removed if there is no candidate inside the search volume. The process is repeated for the next time step with a higher order prediction function [red and black dash line arrows in Fig. 2(a)]. A unique four frame solution is expected for flows with low velocity and acceleration gradients or low particle concentrations. When more than one

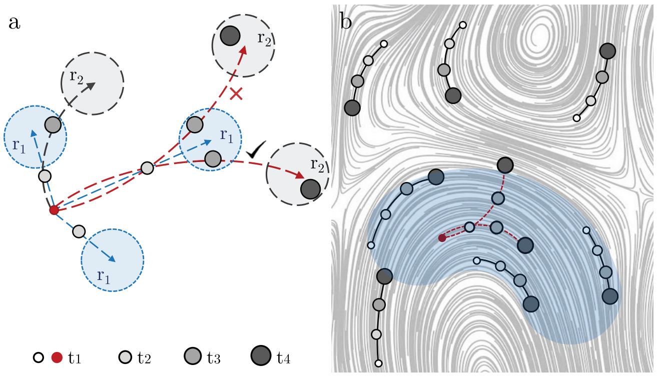

FIG. 2. Schematic view of the LCTI algorithm when two possible four frame solutions exist. (a) LCTI four frame algorithm considering all possible neighbor candidates at t_2 followed by linear predictions (blue dash line arrows). Candidate matching at time step t_3 inside first search volume (blue circle r_1). Second order prediction (red and black dash line arrows) to match possible candidates at t_4 inside second search volume (gray circle r_2). (b) Coherency check between two possible track matches and neighbor coherent motion.

solution exists, LCTI selects the most coherent track to solve the ambiguities, as shown in Fig. 2(b). Otherwise, a particle can spatially meet a group of other particles with no coherency link between them. We recall here that coherent refers to a group of particles that are having the same Lagrangian behavior spatially and temporally. A function is therefore required to determine the coherent and non-coherent clusters of particles locally. More details and principles on the coherent motion of particles are discussed in Sec. II C.

C. Coherent track detection

Recently, Lagrangian Coherent Structures (LCS) have been applied in PIV/PTV experiments for flow structure analyses.[17–20] However, previous studies have not yet combined the LCS extraction with the velocimetry algorithms and mostly focused on using LCS as a post-processing tool, to the best of our knowledge. Several methods have been proposed to identify LCS by looking for separatrix regions in time.[13,21] Separatrices exist in boundaries (i.e., ridges) between different structures. A schematic view of the boundaries between vortices in a 2D isotropic homogeneous turbulent flow is shown in Fig. 3. Multi-clusters of particles spatially exist in the vicinity of the target particle (the dark blue particle in Fig. 3). All red and blue particles are neighbors of the target particle. However, the trajectories of each colored cluster temporally evolve in separated directions. LCS can be used to determine if a spatially neighbor particle is coherent or non-coherent over a temporal scale.

Suppose the flow is dominated by coherent structures such as in a 2D isotropic homogeneous turbulence illustrated in Fig. 3, the global LCS analysis can extract meaningful boundaries between structures. Difficulties in interpretation arise, however, when the flow carries 3D complex motions and numerous local structures. We, therefore, suggest local coherent structure extractions instead of a global calculation for which only coherent clusters and boundaries over neighbor trajectories are computed. Therefore, the complexity of the global LCS view is simplified into a small number of clusters around the target particle,

Published under an exclusive license by AIP Publishing

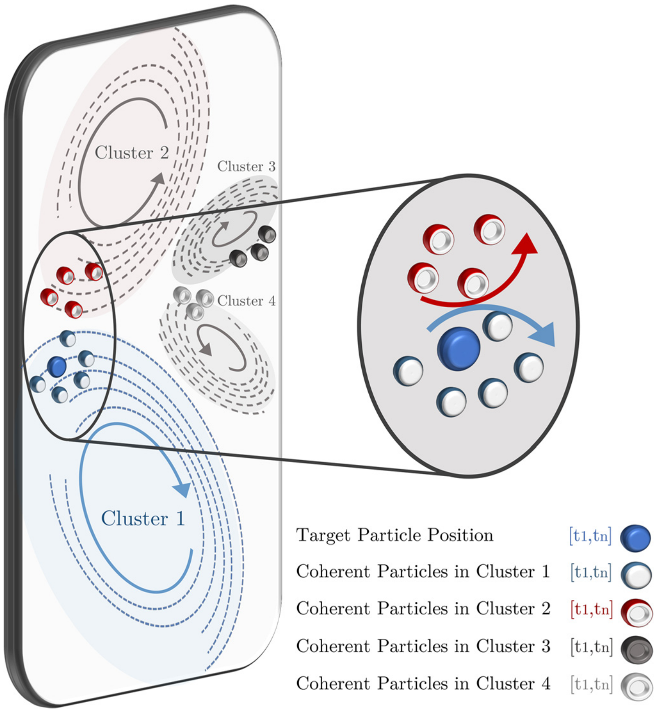

FIG. 3. 2D schematic of particle motions inside vortices. Each color belongs to a group of coherent clusters. The target dark blue particle with coherent neighbor particles is located in a clockwise vortex (blue cluster), while non-coherent particles belong to different clusters. The target particle is non-coherent with neighbor particles in the red cluster.

such as in Fig. 3. In the local view, the curve or surface boundaries divide the local spatial area into discrete regions with different dynamic motions,[22] and motions across these boundaries are negligible.[23] Furthermore, the LCS boundaries can move, evolve, and vanish in spatial space as the flow pattern changes temporally.

In the local Lagrangian frame, separatrices can be obtained from Finite Time Lyapunov Exponent (FTLE) by measuring the amount of stretching between the target particle and its neighbor particles over finite time.[24,25] Raben *et al.*[25] showed that the normalized average error and normalized root-mean-squared (RMS) error of the FTLE map decreases with increased particle concentration. This trend is favorable because ongoing PIV/PTV experiments consistently succeed in achieving higher particle concentrations. Meanwhile, it is less likely to have ambiguities due to multi-possible solutions in low particle concentration cases. As a result, there is no critical need for the coherency check in low particle concentration cases.

As discussed in Sec. II B, if a possible track is coherent with its neighbor tracks, it will be indexed into the tracked poll. To compute FTLE, we assumed that the local flow map is the particle trajectory in finite time from t_0 to t_0+T (in this study $T \geq 4$). The idea is to analyze the spatial displacements between the target particle and its neighbors. The flow map of a single particle can be formulated as

$$\emptyset_{t_0}^{t_{0+T}}(x) : x(t_0) \rightarrow x(t_0+T), \tag{1}$$

where $x(t_0)$ is the starting position of the interval time T, and $x(t_0+T)$ is the final position. Difference between the flow maps of the target particle $\emptyset_{t_0}^{t}(x_0^p)$ and its neighbor $\emptyset_{t_0}^{t}(x_0^n)$ would result in a vector displacement as follows:

$$\delta x(t) = \emptyset_{t_0}^{t}\left(x_0^p\right) - \emptyset_{t_0}^{t}\left(x_0^n\right). \tag{2}$$

This vector displacement contains transformation between the initial and final positions of two particles. Equation (2) can be linearized by using the first term of the Taylor series of $\emptyset_{t_0}^{t}(x_0^n)$ expanded about x_0^p,

$$\delta x(t) \approx \frac{\partial \emptyset_{t_0}^{t}\left(x_0^p\right)}{\partial x_0}\delta x_0, \tag{3}$$

where $\delta x_0 = x_0^p - x_0^n$. The state-transition matrix $\partial \emptyset_{t_0}^{t}(x_0^p)/\partial x_0$ is also known as deformation gradient tensor $\nabla \emptyset_{t_0}^{t}(x_0)$.[23] The deformation tensor carries valuable information including the rate of expansion, compression, and rotation. The magnitude of $\delta x(t)$ is

$$\begin{aligned} |\delta x(t)| &= \sqrt{\delta x(t)\delta x(t)} \\ &= \sqrt{\left(\nabla \emptyset_{t_0}^{t}(x_0)\delta x_0\right)\left(\nabla \emptyset_{t_0}^{t}(x_0)\delta x_0\right)} \\ &= \sqrt{\delta x_0\left(\nabla \emptyset_{t_0}^{t}(x_0)\nabla \emptyset_{t_0}^{t}(x_0)\right)\delta x_0}, \end{aligned} \tag{4}$$

where we define

$$\Delta = \nabla \emptyset_{t_0}^{t}(x_0)\nabla \emptyset_{t_0}^{t}(x_0). \tag{5}$$

Δ is a symmetric positive definite matrix, also known as the right Cauchy–Green deformation tensor[23] with three real and positive eigenvalues in a 3D domain over finite time.

As mentioned before, FTLE measures the rate of stretching between the target particle and its neighbor. The maximum eigenvalue of the Cauchy–Green tensor $\lambda_{max}(\Delta)$ shows the expansion or separation in finite time. Furthermore, the eigenvector corresponding to $\lambda_{max}(\Delta)$ represents the direction of the separation. Eventually, the magnitude of the maximum displacement can be written as

$$|\delta x(t)_{max}| = \sqrt{\delta x_0 \lambda_{max}(\Delta)\delta x_0} = \sqrt{\lambda_{max}(\Delta)}|\delta x_0|, \tag{6}$$

and the FTLE value $\Lambda_{t_0}^{t}$ is defined as

$$\Lambda_{t_0}^{t} = \frac{1}{|T|}\sqrt{\lambda_{max}(\Delta)} = \frac{1}{|T|}\log\left(\frac{\delta x(t)}{\delta x(t_0)}\right). \tag{7}$$

A lower FTLE value means the neighboring particle is acting similarly, with no sign of separation with the target particle over the finite time. High values in the FTLE field show the existence of ridges that divide the local area into different clusters of coherent particles. With this formulation, it is possible to index a group of neighbor particles as coherent or non-coherent with the target particle. As we discussed in Sec. II B, the LCTI algorithm checks if the Lagrangian coherency is valid for each possible four frame tracks to avoid non-coherent reconstructions. Assuming two possible matches exist for the target particle [see Fig. 2(b)], we start by fitting a smooth curve over each known neighbor tracks to reduce the noisy reconstruction effect on the coherency detection. Then LCTI locally computes the FTLE map over the fitted tracks without considering two possible matches. If the FTLE map shows local separations, neighbor tracks in the same cluster with the target particle are classified as coherent neighbors.

Published under an exclusive license by AIP Publishing

As illustrated in Fig. 3, the local region is divided into two blue and red clusters. Only the neighboring tracks inside the blue cluster are coherent with the target particle. All neighbor tracks are coherent neighbors if no separation is detected. To quantify a threshold for the FTLE ridge detection, we assessed the FTLE map for the case of 2D homogeneous isotropic turbulence given by Direct Numerical Simulation (DNS). We found that values above the threshold 0.25 are optimal criteria to estimate the FTLE ridge positions. This threshold was in agreement with studies using global FTLE ridge calculation in the range of $50\% - 80\%$ of the maximum FTLE value.[26] There are valuable studies in ridge detection algorithms with extensive computation costs that can be employed instead of using a constant FTLE threshold (see, e.g., Shadden *et al.*[23]). After the coherent neighbor determination, LCTI checks the FTLE value for each possible match and neighbors. Finally, the most coherent match with the coherent neighbors will be indexed. This process continues iteratively until no track is found to be coherent with the tracked poll.

III. SYNTHETIC EVALUATION

We first applied the LCTI algorithm to synthetic data as an effective tool with three specific objectives including, algorithm performance assessment, algorithm sensitivity analysis as a function of characteristic parameters, and algorithm comparison.[27] This approach has been utilized widely in recent algorithm developments of PIV/PTV studies.[7,16,28,29] In this section, we consider a set of synthetic data for the wake flow downstream of a smooth cylinder. We first describe the creation of the Lagrangian data by transporting the synthetic particles in the Eulerian velocity volume. After that, we discuss how to set characteristic parameters for the synthetic data, including (1) particle concentration, (2) temporal scale, and (3) noise level. We also address the accuracy analyses of LCTI in comparison with other schemes, followed by the sensitivity analysis of LCTI to every characteristic parameter. Finally, we discuss the LCTI performance in the LPT challenge for the wall-bounded wake flow behind the cylinder.[15]

A. Synthetic data creation

1. Particle transport

An open-source Direct Numerical Simulation (DNS) code, named Incompact3d, was employed to simulate the wake downstream of a smooth cylinder at a Reynolds number equal to 3900. Incompact3d is part of the Xcompact3d framework dedicated to the study of turbulent flows using supercomputers,[30] and it is dedicated to the incompressible Navier–Stokes equations. It is based on sixth-order finite-difference schemes on a Cartesian mesh for spatial discretization and, for the present simulation, a third-order Adams–Bashforth scheme for the time advancement. The main originality of Incompact3d is that the Poisson equation for the incompressibility of the velocity field is fully solved in spectral space via the use of relevant 3D Fast Fourier transforms (FFTs). With the help of the concept of modified wavenumber, the divergence free condition is ensured up to machine accuracy. The pressure mesh is staggered from the velocity one by half a mesh to avoid spurious pressure oscillations observed in a fully collocated approach. The simplicity of the mesh allows an easy implementation of a 2D domain decomposition based on pencils. More information of the code can be found in Laizet and Lamballais,[31] Laizet and Li.[32] In the present work, the smooth cylinder is modeled using a customized immersed boundary method with an artificial flow inside the cylinder to ensure the smoothness of the velocity field while imposing a no-slip boundary condition at the cylinder. Inflow/outflow boundary conditions are implemented along the streamwise direction with free-slip and periodic boundary conditions along the vertical and spanwise directions, respectively. Comparisons with experiments for this test case can be found in Chandramouli *et al.*[33] A DNS performed with high-order schemes is the natural choice for the present work, with the highest possible spatial and temporal accuracy for Eulerian/Lagrangian data. Velocity and pressure Eulerian data are stored as well as Lagrangian trajectories for a duration of three vortex sheddings. These data will be made in open access and available to the public.

Lagrangian particle transport is a process to extract trajectories from an Eulerian velocity volume.[34] In this study, the Eulerian velocity volume is a fraction of flow around the smooth cylinder of diameter D, starting from $1D$ upstream of the cylinder center to $3D$ downstream in the streamwise direction to reduce the computation costs and only focus on the most complex flow regions. Every DNS snapshot (three velocity components and pressure field) requires roughly 6 GB of storage. Eulerian data were only recorded every 10 DNS time steps due to storage limitation. To quantify the accuracy of particle transport, we compared trajectories obtained from every 10 with every 1 DNS time steps in a reduced domain. It was found that the mean of position error after 1000 DNS time steps is 3.28 times larger than the Kolmogorov length scale η. The standard deviation of position error was $\sigma_{STD} = 0.017\,\eta$. It means that the trajectories we built are not exactly ground truth due to small position deviation compared with every 1 DNS time step. However, it has no impact on the following assessment of the method we propose. More details of the current synthetic data can be found in Khojasteh *et al.*[35] As mentioned in Table I, the domain of interest $L_x \times L_y \times L_z$ has a dimension of $4D \times 2D \times 2D$ (from $1D$ upstream the cylinder). The original computational domain $20D \times 20D \times 6D$ is discretized with $n_x \times n_y \times n_z = 1537 \times 1025 \times 256$ mesh nodes with a time step of $0.000\,75D/U_\infty$ (where

TABLE I. DNS domain specification.

Domain	Dimension L_x	Dimension L_y	Dimension L_z	Grid size nx	Grid size ny	Grid size nz	Time step dt
Computation domain	20D	20D	6D	1537	1025	256	$0.000\,75D/U_\infty$
Domain of interest	(4–8)D	(9–11)D	(2–4)D	308	328	87	$0.0075D/U_\infty$
Cylinder location	5D	10D	(0–6)D				

Published under an exclusive license by AIP Publishing

U_∞ is the freestream velocity). The mesh is uniform in the streamwise and spanwise directions while a non-uniform mesh is used in the vertical direction, with a mesh refinement toward the center of the cylinder. The finest mesh size in the vertical direction is $\Delta y_{\min} = 0.005\,63D$. Data are saved for 1500 snapshots every 10 DNS time steps dt_{DNS} corresponding to a duration of nearly three vortex sheddings. From these data, particles are transported according to the following steps: First, synthetic random particles are created in the domain of interest for the first time step; second, accurate time stepping and spatial interpolation schemes are used to transport scattered random particles in time and space.[34] We estimated the Lagrangian particle velocities by spatial trilinear interpolation from 8 nearest Eulerian velocities at each time step. The synthetic particle positions are updated in time using the fourth order Runge–Kutta scheme. Finally, we collected the ground truth Lagrangian trajectories by repeating the transport process for each time step.

2. Particle concentration

In most PIV/PTV studies, particle concentration refers to the number of particles per pixel (ppp), which is principally an image-related parameter. The relation between the particle concentration and the turbulence length scales determines the maximum achievable spatial resolution. Therefore, it is crucial to address the number of particles corresponding to Kolmogorov (η), Taylor (l), and Integral (D) length scales. To quantify these relations, we used two volumetric metrics, namely, particles per cubic Kolmogorov scale (ppη^3) and particles per cubic integral scale (ppD^3), instead of ppp. We chose three low, moderate, and high particle concentrations, respectively, equal to 2000, 20 000, and 200 000 particles (see Table II). It is possible to assess cases with more particles and reach the particle concentration up to the DNS spatial resolution numerically, but very few PTV experimental studies have reached more than 200 000 particles in practice due to the PTV seeding and the 4D-PTV limitations. One limitation can be the IPR reconstruction that fails for higher particle concentrations,[2] and therefore the whole 4D-PTV process diverges. Figure 4 shows the turbulence energy spectrum marked by the three particle concentrations (blue lines) compared with the turbulence length scales (red dash lines). Referring to the DNS data in Sec. III A, we estimated the Kolmogorov length scale as $D/\eta \sim Re^{3/4}$ that is almost 2.8 times smaller than the average mesh size in the y-direction. Since the cylinder is inside our domain, it is necessary to subtract the volume of the cylinder before computing ppη^3. Accordingly, the spatial resolution for the case with 2000 particles leads to unresolved Taylor and Kolmogorov scales. However, there are enough particles to resolve the large flow motions. Taylor length scales can be resolved by adding an order of magnitude more particles, but the smallest scales are still unresolved with 10^{-4} ppη^3. As shown in Fig. 4, in our case, even 200, 000 particles with 10^{-3} ppη^3 are not enough to resolve the smallest turbulence length scale at Reynolds number equal to 3900.

TABLE II. Synthetic particle concentration selection in terms of particles per cubic Kolmogorov and integral scales.

Case	Particles	ppη^3	ppD^3
Low	2000	10^{-5}	139
Moderate	20 000	10^{-4}	1386
High	200 000	10^{-3}	13861

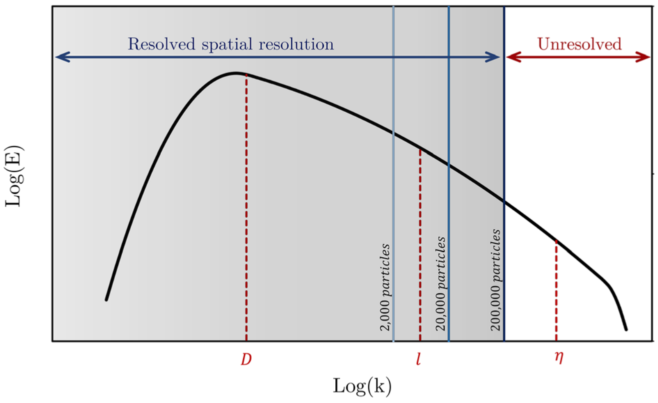

FIG. 4. Schematic view of energy spectrum in turbulence. Resolved and unresolved spatial resolutions of 2000, 20 000, 200 000 particles in blue lines with respect to integral (D), Taylor (l), and Kolmogorov (η) length scales in red dashed lines.

3. Temporal scale

Schanz *et al.*[1] computed the temporal scale as a function of the original experiment time sampling rate, resulting in a mean 3D particle displacement of around 6 pixels for the synthetic data analysis. However, the temporal scale selection requires satisfying the real experiment condition and should be characterized by the flow physics. We defined the temporal resolution as a ratio of turbulence time scales. The non-dimensional form of the temporal scales can be written as dt/dt_{DNS}, dt/τ_η, and dt/T_D, where dt is the temporal scale for either synthetic or experiment study, and dt_{DNS}, τ_η, and T_D are the DNS, Kolmogorov, and integral time scales, respectively. By defining $T_D = D/U_\infty$, we estimate the Kolmogorov timescale from ratio of the largest to smallest time scales as $T_D/\tau_\eta \sim Re^{1/2}$. To mimic the real experiment condition, we gathered similar wake flow studies (mainly cylinder wake flows) and plotted the relations of dt/τ_η with their Reynolds numbers in Fig. 5. It is shown that low Reynolds number experiments can resolve the Kolmogorov timescale.[36] The achievable temporal scale increases with the Reynolds number, mainly because the data acquisition frequency limits the experiments with the order of $1-3$ kHz for the majority of studies mentioned in Fig. 5. In our synthetic case, the DNS time step is roughly 20 times smaller than the Kolmogorov timescale. Such a case with every 10 DNS time step ($dt/\tau_\eta = 0.47$) can resolve the Kolmogorov timescale, but it is unlikely to achieve such a high acquisition rate in practice, particularly when the fluid is air. Particle trajectories are very smooth with small displacements in this case. However, according to Fig. 5, the temporal scale dt/τ_η stays relatively large, between 2.5 and 5 for studies close to a Reynolds number of 3900 that is approximately equal to every 50 and 100 DNS time steps. As listed in Table III, we chose three low, moderate, and high time steps starting from every 10, 50, and 100 DNS time steps. It is worth mentioning that it takes 1333 and 6667 DNS time steps to reach one integral timescale and one vortex shedding, respectively.

Published under an exclusive license by AIP Publishing

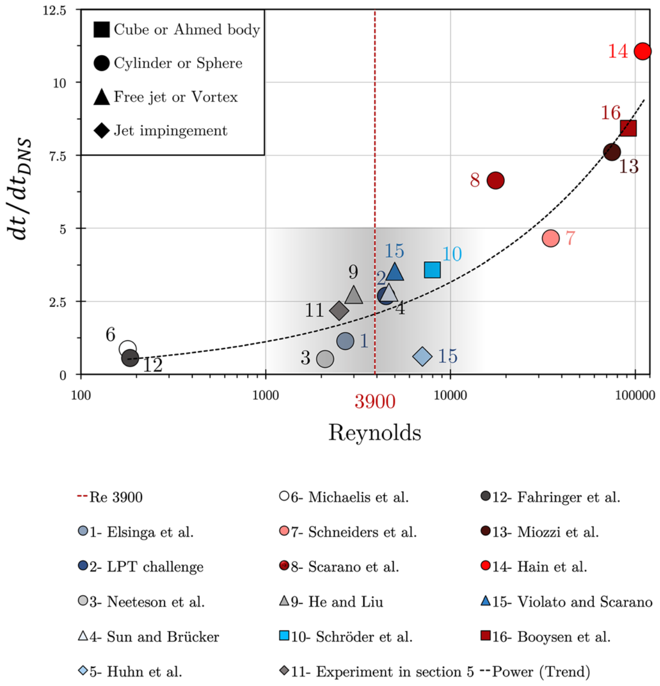

FIG. 5. Synthetic timescale selection with respect to recent similar wake flow experimental studies and different Reynolds numbers. Each symbol represents a family of flow configuration.[15,36–49]

4. Noise ratio

In this paper, we created noise (i.e., false particles) in the vicinity of true positions. A false particle is randomly distributed around the true position with a maximum displacement radius. Noise ratio (NR) of 0.1 means 10% of true particles at every time step has false particles in their vicinities. A false particle around a single track also impacts the track detection accuracy for other neighbor tracks, particularly in dense and intersection situations. In this study, we created three noise ratios, 0, 0.1, and 0.2.

B. Evaluation and sensitivity analyses

In this section, the performance of the LCTI is assessed and compared against ETI and 4BE-NNI methods. Quantitative accuracy and sensitivity analyses are performed for different particle concentrations, temporal scale, and noise ratio compared to the ground truth trajectories (see Sec. III A). After the initialization step, the trajectory of each particle is classified as either untracked, wrong, or true track. A dominant number of true tracks would ease the 4D-PTV process to converge within short time steps. It is worth mentioning that a 4D-PTV process is converged if all particles inside the domain are tracked.

TABLE III. Synthetic time step selection in terms of DNS, Kolmogorov, and integral time scales.

Case	dt/dt_{DNS}	dt/τ_η	dt/T_D
Low	10	0.47	0.01
Moderate	50	2.34	0.04
High	100	4.68	0.08

Figure 6 compares the ratio of detected true tracks obtained from LCTI, ETI, and 4BE-NNI techniques with different characteristic parameters of ppη^3, dt/τ_η, and NR. We assessed each technique based on $3 \times 3 \times 3$ scenarios representing low, moderate, and high levels of each characteristic parameter (see Tables II and III). The 3D view of particle trajectories for the current assessment is shown in Fig. 12(c) (Multimedia view). All three techniques performed equally well for low particle concentration and small temporal scale by reconstructing over 95% of true tracks [see Fig. 6(a)]. Tracking is not difficult under such scenarios characterized by small particle displacement with relatively large spatial distance between neighbor tracks. By increasing dt/τ_η in low particle concentration cases, the ratio of detected true tracks drops down to approximately 90% and 75% in ETI and 4BE-NNI techniques, respectively, whereas LCTI remains stable. The large relative distance between neighbor trajectories due to low particle concentration reduces the ambiguity in finding possible tracks. Results of low particle concentration cases for all three techniques show that the 4D-PTV process has to recover less than 25% of remaining untracked particles to converge in the worst case; thereafter, a short convergence time is expected. The ratio of true tracks drops linearly by increasing the noise ratio (NR) for all techniques with approximately the same order of magnitude. We found that the drop in the detected true tracks caused by the noise ratio (NR) is nearly independent of the other two characteristic parameters. Comparing the three characteristic parameters reveals that the temporal scale has the most deterministic impact on the detected true tracks for all techniques. If dt/τ_η stays low, all techniques can cover over 95% of true tracks regardless of the noise ratio and the particle concentration. Low temporal scale value means that particle displacements are very small. Even two frame nearest neighbor techniques can reconstruct the majority of true tracks. However, in more realistic conditions, when the temporal scale dt/τ_η is high, LCTI performed significantly better than the other two techniques. Particle concentration also plays an essential role in the initialization performance. The ratio of detected true tracks for LCTI stays consistently above 95% in particle concentrations of 10^{-4}ppη^3 [see Fig. 6(b)]. In a worse scenario, when the particle concentration is 10^{-3}ppη^3 associated with high temporal scale, LCTI still can recover over 75% of true tracks that is considerably higher than ETI and 4BE-NNI with roughly 50% and 25% true tracks, respectively [see Fig. 6(c)].

The synthetic data evaluation showed that LCTI systematically outperforms other competing techniques. However, it should be mentioned that the cost of this achievement is expensive because it computes the coherency for every possible tracklet. We found that LCTI requires roughly four times more computation time on a single CPU (central processing unit) core than a classic four frame based initialization technique without any post-treatment. To this end, an appropriate initialization technique should be chosen depending on the measurement condition. As an example, there is no need to perform a sophisticated initialization technique if the temporal scale (i.e., sampling rate) and the particle concentration are low in comparison with the turbulence scales. It is important to note that using sophisticated initialization techniques such as LCTI is crucial to prevent 4D-PTV

Published under an exclusive license by AIP Publishing

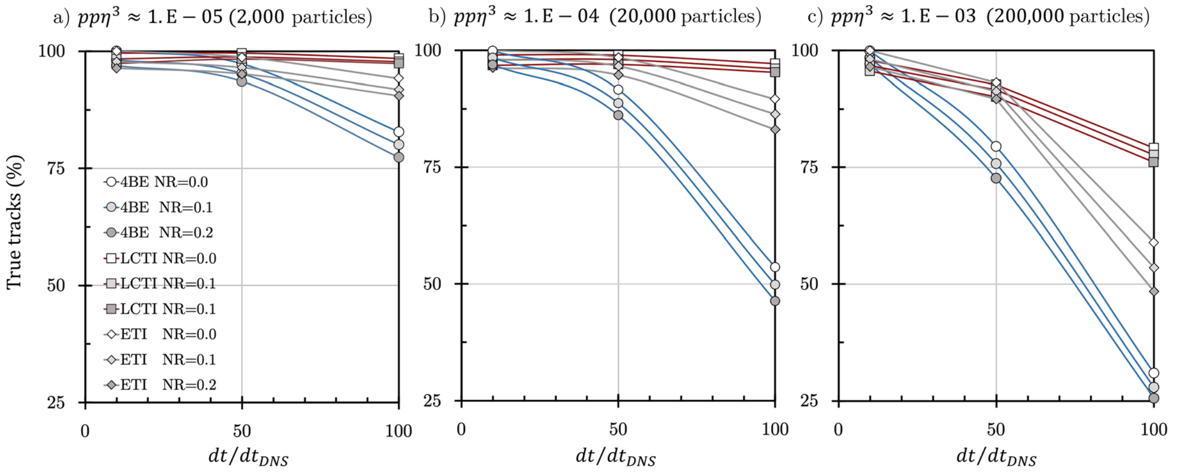

FIG. 6. Comparison performances of three track initialization techniques in terms of fraction of true particle detection by changing each characteristic parameter. Particle concentration is increasing from (a) to (c).

failure in challenging cases with a large temporal scale and high particle concentration. Apart from the initialization performance based on the number of true tracks, we performed further parametric analyses on each technique to determine how untracked and wrong trajectories are sensitive to the characteristic parameters. We started LCTI from a base case with 10^{-4} $pp\eta^3$ particle concentration, $dt/\tau_\eta = 2.34$, and zero noise ratio. Afterwards, we increased each parameter separately until the fraction of true tracks drops down to 80%. The remaining 20% is a mix of untracked and wrong trajectories. Then we performed the same parameters on 4BE-NNI and ETI techniques. Lagrangian flow maps for increasing each characteristic parameter are shown in Fig. 7. If we increase the temporal scale, both untracked and wrong

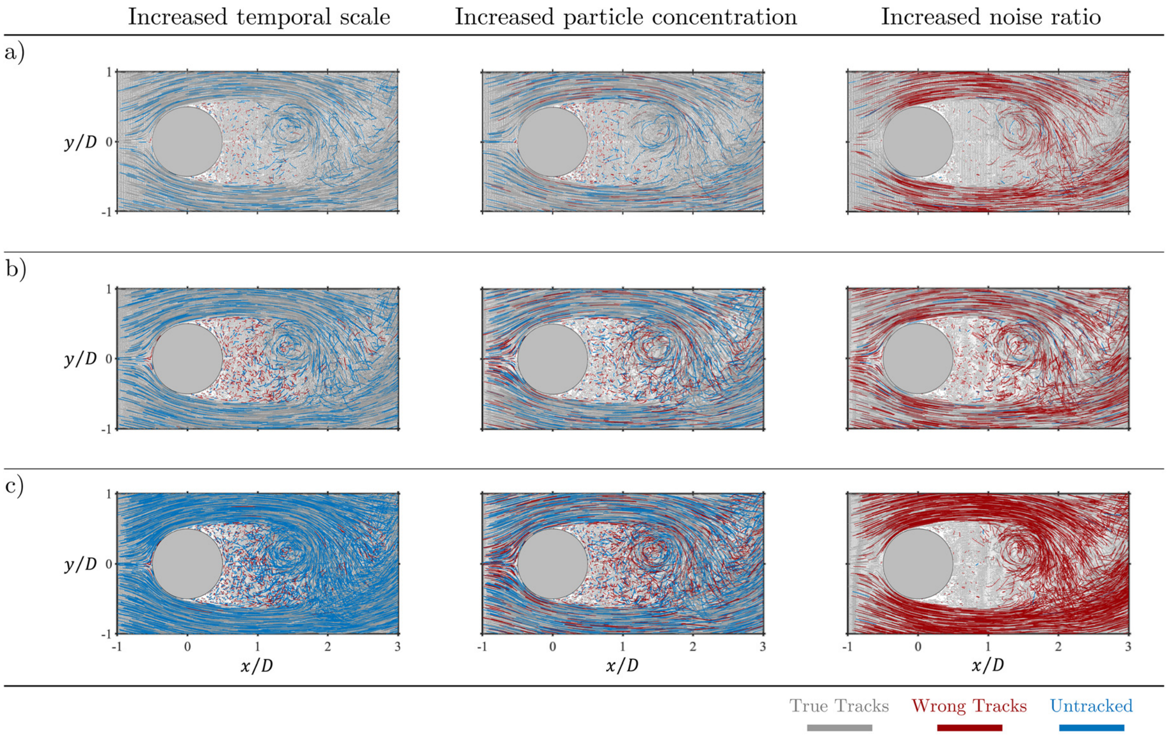

FIG. 7. Sensitivity analysis of (a) LCTI, (b) ETI, and (c) 4BE-NNI compared with DNS ground truth by increasing temporal scale, particle concentrations, and noise level from top to bottom. Tracks in blue and red color represent untracked and wrong trajectories, respectively. Gray tracks are true trajectories that the algorithm built (numbers of gray tracks are scaled down for having a clear view of red and blue tracks).

Published under an exclusive license by AIP Publishing

tracks increase as the number of true tracks drops. Under such a scenario, the particle displacements are large and comparable with local distances between the neighbor particles. Therefore, less information between two-time steps is available, which drastically increases the possibility of having untracked particles. Depending on the region of the flow, the majority of untracked trajectories (blue color in Fig. 7) exist around the two high shear sides of the wake region. Although the number of untracked trajectories dominates the whole Lagrangian flow map, more wrong tracks (red color in Fig. 7) than untracked trajectories are observed inside the wake region. By increasing the particle concentration, both untracked and wrong trajectories raise homogeneously with nearly the same proportion through the Lagrangian flow map for all three techniques. Interestingly, the number of wrong tracks is still comparably larger than the number of untracked particles inside the wake region. It shows that regional flow behaviors can have a direct impact on the initialization performance. The impact of increasing the noise ratio is also shown in Fig. 7. We found a dominated number of wrong tracks in all regions with increased noise ratio. In this study, we created the noise (i.e., false particles) in the vicinity of true particles that causes more initialization ambiguities. Due to this reason, LCTI yields more wrong trajectories with increased noise ratio. Besides, wrong indexing of a track takes away at least one true or false particle that may propagate the wrong detection to another neighbor track too. In practice, for 4D-PTV, wrong initialized tracks increase the failure risk and need to be appropriately eliminated in a subsequent prediction-optimization step. The untracked particles, on the other hand, require more iteration and convergence time.

Sensitivity analysis in Fig. 7(a) implies that LCTI is likely to have more wrong tracks inside the wake region with increased noise ratio, particle concentration, and temporal scale. However, different behaviors have been seen depending on the characteristic parameters in overall. The high turbulence intensity level at Reynolds 3900 creates small flow structures inside the wake region. Consequently, particles inside this region are coherent with a small number of neighbors and quickly change to different coherent clusters. This behavior brings more complexity for the coherency detection that might be the reason for having a dominating number of wrong tracks, despite relatively small particle displacements. Schanz *et al.*[51] observed a similar issue when particles of a specific region move slowly compared to the rest of the domain. One approach for improving trajectory in such regions is performing temporal filtering schemes by adjusting the temporal scale to track the slowest particles.[51] Comparing the LCTI sensitivity with the other two techniques shows that both 4BE-NNI and ETI have similar behaviors to each characteristic parameter with higher proportions of untracked and wrong trajectories [see Figs. 7(b) and 7(c)]. The reason for having higher mix of untracked and wrong trajectories can be found in Fig. 6. A drop in the number of true tracks with increased characteristic parameters leads to more untracked and wrong trajectories, which is in agreement with findings of Fig. 7.

C. LPT challenge

LCTI was implemented into KLPT[3] to run the whole 4D-PTV process (see Fig. 1). KLPT featured by LCTI (KLPT-LCTI) was examined on the time-resolved data from the LPT challenge[15,50] at four particle densities (i.e., concentrations) from 0.005 up to 0.08 ppp. The challenge cases were obtained from the wall-bounded wake flow behind a cylinder at a momentum thickness Reynolds number Re_θ of around 4500. In terms of turbulence length scales, particle concentration of the mentioned four cases varied between 2×10^{-7} and 3×10^{-6} ppη^3. The domain of interest was set at $100 \times 50 \times 30\,\mathrm{mm}^3$ downstream of the cylinder. The image acquisition rate was equal to 600 μs, resulting in $dt/\tau_\eta = 2.68$ temporal scale. At the lowest ppp, the proposed method managed to reconstruct over 99% of true particles accurately. Percentage of true particles maintained over 99% for higher densities (i.e., 0.025, 0.05, and 0.08 ppp). Accordingly, missed and ghost particles were less than 1%. The case studies of the LPT challenge revealed that the positional Root-Mean-Square error (RMSE) of KLPT-LCTI increased linearly with ppp, but it remained below 0.0041 mm for all four particle densities. This illustrates the reliable performance of the LCTI at particle densities lower than 0.08 ppp, knowing that most of the 4D-PTV real experiments perform at 0.05 ppp particle density or lower.

For densities higher than 0.08 ppp, a more accurate initialization technique could prevent the 4D-PTV algorithm from failing or improve its convergence speed. We highlighted that KLPT featuring LCTI succeeded in reconstructing tracks at the density of 0.12 ppp, while KLPT featuring NNI failed to converge. Figure 8 shows an example of coherent motion detection by LCTI at the density of 0.12 ppp. The particle trajectories obtained from the proposed method are shown in Fig. 12(a). Questions have been raised about the 4D-PTV sensitivity to the number of initialized particles at the beginning. We illustrated this issue in the LPT challenge case with 0.12 ppp and over 120 000 particles. As shown in Fig. 9, the KLPT-LCTI process reaches no more than 85 000 (i.e., 70%) final tracks if the process starts with any number below 30 000 initialized tracks. However, starting with 60 000 initialized tracks leads to cover over 99% of final trajectories after 30 time steps at 0.12 ppp. The evidence from this study indicates that the number of initialized tracks is one deterministic contributor to the 4D-PTV convergence at high-density scenarios. Without a proper track initialization algorithm, a 4D-PTV scheme would not be able to recover the majority of tracks eventually.

IV. EXPERIMENT DEMONSTRATION

To demonstrate the potential of the LCTI in a practical configuration, we used the data from a volumetric experiment of liquid–liquid jet impingement on a circular wall at a Reynolds number equal to

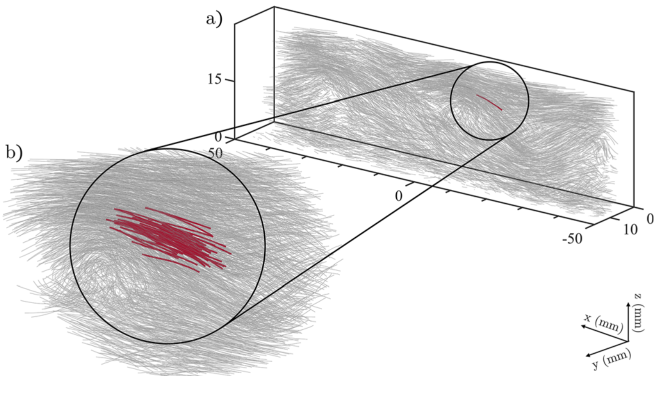

FIG. 8. LCTI trajectory results for the LPT challenge wake flow at 0.12 ppp:[15,50] (a) slice view in y direction of particle trajectories in gray and the target track in red and (b) cluster of coherent tracks with the target track in red.

Published under an exclusive license by AIP Publishing

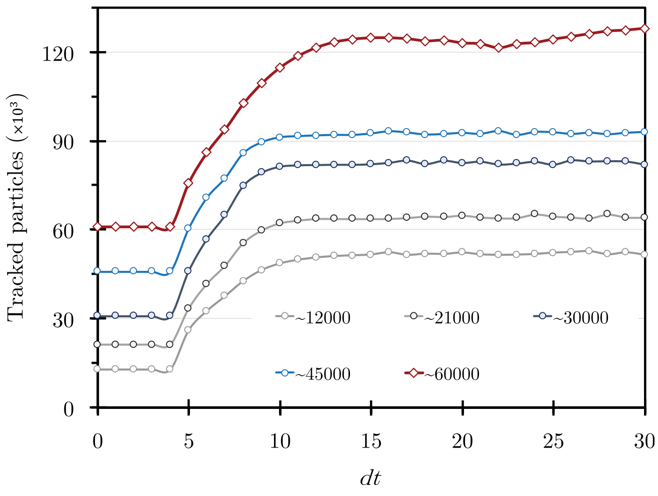

FIG. 9. Sensitivity of the 4D-PTV convergence to the number of initialized tracks for the LPT challenge high-density case at 0.12 ppp over first 30 time steps. Number of initialized tracks after the first four frames varies from ∼12 000 to ∼60 000.

2500. Perpendicular impinging the jet into the wall creates significant deceleration inside the jet core. The deceleration and directional 3D motions of particles are featured by multi-vortex rings around the jet and secondary vortex structures after the impingement. Figure 10 shows the schematic view of the experiment setup. Four Phantom M310 cameras with 1200×800 resolution and the maximum frequency of 3.26 kHz empowered (see Table IV). Nikon 105 mm macro F2.8 (aperture was set to F22) lenses were adjusted by the double angle Scheimpflug adaptors. As shown in Fig. 10, two cameras were positioned in 24° with forward scatter light, and the other two cameras were in backward scattering at 13°. 15 mJ LDY 300 laser and a mirror at the bottom of the water tank were set for the illumination with

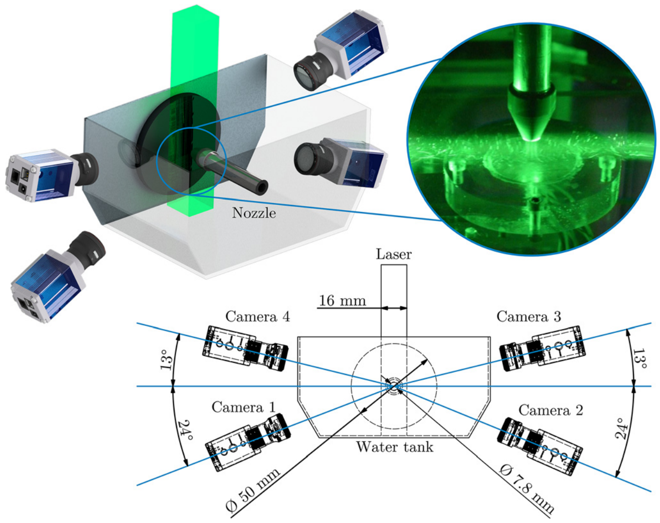

FIG. 10. 3D schematic view of the jet impingement experimental, with snapshot of the experiment in zoom balloon.

TABLE IV. Experiment setup parameters.

Cameras	4× Phantom M310
Lenses	4× Nikon 105 mm
Illumination volume	$16 \times 51 \times 35\ \text{mm}^3$
Resolution	1200×800
Pixel size	20 μm
Acquisition frequency	0.5 kHz
Exposure time	250 μs
Particle concentration	0.03 ppp
Number of images per experiments	2000 frames

0.5 kHz frequency ($dt/\tau_\eta = 2.18$) and converted into the rectangular light volume. The measurement volume was $16 \times 51 \times 35\ \text{mm}^3$ ($29\ \text{cm}^3$). The seeding particles were hollow glass spheres with $9 - 13\ \mu$m diameter and $1.1\ \text{g/cm}^3$ density. The particle concentration was approximately 0.03 ppp that was equivalent to $2 \times 10^{-6}\ \text{pp}\eta^3$. The calibration error was less than 0.03 pixel. The measurement uncertainty due to the displacement error and the global bias errors was estimated to be in $2.0 - 3.5\%$ of the boundary layer in the wall jet region. More details in this experiment can be found in Yang and Heitz.[3]

In this section, we performed the 4D-PTV process without prediction and optimization parts to particularly demonstrate the LCTI performance. Therefore, the reconstructed particle positions with 1 pixel allowed triangulation error by IPR were employed in 20 consecutive time steps followed by multi four-frame LCTI processes. Trajectory results obtained from LCTI are shown in Figs. 11 and 12(b). Away from the jet impingement region, the trajectories are relatively smooth with small displacements. As explained earlier in Sec. III A, there is no critical issue in such regions, and a simple initialization technique can track the majority of particles. Small trajectories away from the jet, therefore, are omitted in Fig. 11 to concentrate on the most complex regions. As the jet impinges on the wall, trajectories decelerate highly and turn alongside the wall, but still tend to keep their coherent local motions. The proposed method effectively

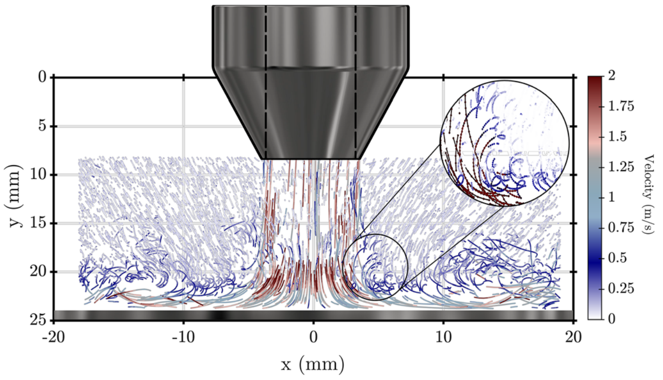

FIG. 11. Side view of the trajectories, colored by their velocity magnitudes at 0.03 ppp. Low-velocity tracks away from the jet core are filtered for clear qualitative view thanks to the color bar.

Published under an exclusive license by AIP Publishing

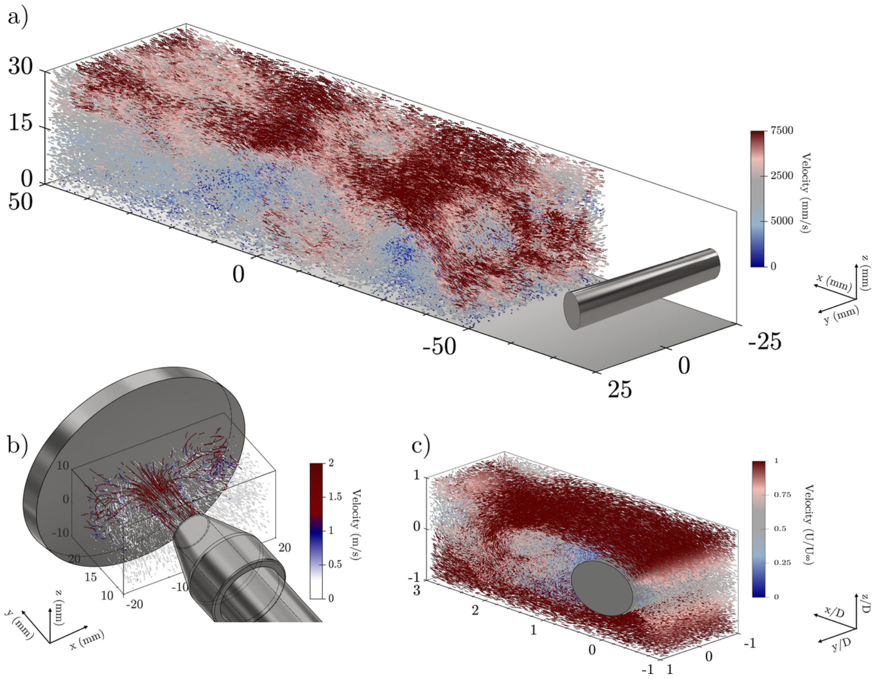

FIG. 12. 3D view of particle trajectories. (a) The LPT challenge downstream trajectories of the wall bounded flow in the wake of a cylinder at ppp = 0.12. (b) Jet impingement experiment case study. (c) Synthetic case for the wake behind a smooth cylinder. Multimedia views: https://doi.org/10.1063/5.0060644.1; https://doi.org/10.1063/5.0060644.2; https://doi.org/10.1063/5.0060644.3

detected these coherent trajectories although complex behaviors exist. We found that the trajectories around the jet tend to circulate (see the zoom balloon in Fig. 11). This reveals strong evidence of the particle coherent motions impacted by the vortex rings. Near wall trajectory reconstructions show signatures of the secondary vortices where particles bounce back. The results of this experiment test case support the idea that each particle can temporally and spatially behave in coherence with a group of other neighbor particles. The second significant finding was that the proposed initialization technique could cover most tracks in complex and high gradient regions associated with 3D directional dynamics.

V. CONCLUSIONS AND OUTLOOK

We proposed a novel technique to reconstruct tracklets from four (or multi) frames by leveraging temporal and local spatial coherency of neighbor tracks. These tracks should be consistent with the neighbor coherent motions bounded by LCS ridges. To assess the LCTI performance in various conditions, we have created an open-access synthetic dataset for the wake flow downstream of a smooth cylinder obtained from DNS at a Reynolds number equal to 3900. Future studies by interested readers should focus on using the current open access Eulerian velocity and pressure data as well as synthetic Lagrangian trajectories for algorithm evaluation purposes. We transported particles using the trilinear spatial interpolation scheme followed by the fourth order Runge Kutta temporal integration. In the current dataset, temporal and spatial scales are reported based on turbulence length and time scales. As a result of the parametric study in Sec. III B, we found that a 4D-PTV process requires more sophisticated initialization techniques if either one of the following conditions is met:

- Particle displacements between two time steps are relatively large (i.e., $dt/\tau_\eta > 2.34$) due to either sparse temporal acquisition frequency or high dynamic gradients of particles (velocity and accelerations).
- The particle concentration is high enough to have a length between neighbor particles with the same order of their trajectory displacement lengths that can be quantified as $ppp\eta^3 > 10^{-3}$ in this study;
- The reconstructed particle field is noisy (NR > 0.2).

Under these conditions, LCTI tends to detect more true tracks than recent ETI and 4BE-NNI techniques. We found that the temporal scale severely impacts the true track detection yielded by any initialization techniques. We also analyzed the proportions of untracked and wrong trajectories. The main results indicate that the noise ratio creates more wrong tracks, the particle concentration homogeneously increases both untracked and wrong tracks, and finally, the temporal scale causes more untracked trajectories. Regardless of the characteristic parameters, we found more wrong trajectories than untracked particles inside the wake region.

We also applied LCTI to the time-resolved dataset in the LPT challenge after integrating LCTI to a 4D-PTV scheme, KLPT. Our KLPT-LCTI scheme has achieved state-of-the-art performance. The positional Root-Mean-Square error (RMSE) of the proposed method stayed between 0.0020 mm and 0.0041 mm for particle densities of 0.005 up to 0.08 ppp.[15,50] LCTI is helpful for high particle density data where the portion of initialized tracks after the initialization stage directly impacts the 4D-PTV's convergence performance. At ppp = 0.12, KLPT featuring a simple nearest neighbor initialization scheme fails to yield any valid results. LCTI, on the contrary, can recover much more tracks from the starting four frames; therefore, it provides a more accurate initialized track field. Besides, LCTI also

Published under an exclusive license by AIP Publishing

contributes to following every frame by bringing more new tracklets (length less than 4) from triangulated particles on residual images into the tracked poll (see Fig. 1). This finding agrees with the synthetic analysis part in Sec. III B showing the importance of having an advanced initialization for dense (i.e., high particle concentration) conditions.

LCTI was then tested on the jet impingement experiment. Although the flow was characterized by complexities such as 3D directional motion and trajectory intersections, LCTI successfully reconstructed the majority of tracks solely on particle fields reconstructed by IPR, without the necessity of further prediction and optimization processes. The trajectory results also showed particles coherent motions in large scale flow motions such as vortex rings, impinging area, and secondary vortices. This comprehensive study has demonstrated that coherency based track initialization is a robust approach to reconstruct tracks even in complex situations.

In conclusion, the proposed technique can be used either as an embedded module for the 4D-PTV process or as a standalone four-frame-based tracker. LCTI showed that additional physics-based information increases the accuracy and robustness of the initialization part. For further investigations, the FTLE function can be replaced with advanced LCS detection algorithms (see, e.g., Filippi *et al.*[52]) or other coherent motion detection techniques such as Coherent Structure Colouring[53] (CSC). More research is needed to understand and track clusters of coherent particles in real experiments.

ACKNOWLEDGMENTS

The authors would like to greatly thank the LPT challenge organizing committee, particularly Dr. Andrea Sciacchitano from TuDelft university, for providing the time-resolved challenge results. A special thanks from the authors is given to Anthony Guibert (a former assistant engineer at INRAE) who conducted the impingement experiment in 2016. The authors would finally like to thank EPSRC in the UK for the computational time made available on the UK supercomputing facility ARCHER2 via the UK Turbulence Consortium (No. EP/R029326/1).

DATA AVAILABILITY

The data that support the findings of the Sec. III are openly available in the Data INRAE at https://doi.org/10.15454/GLNRHK, Ref. 54. The data that support the findings of the Sec. IV are available from the corresponding author upon reasonable request.

REFERENCES

[1] D. Schanz, S. Gesemann, and A. Schröder, "Shake-The-Box: Lagrangian particle tracking at high particle image densities," Exp. Fluids **57**, 1–27 (2016).
[2] B. Wieneke, "Iterative reconstruction of volumetric particle distribution," Meas. Sci. Technol. **24**, 024008 (2013).
[3] Y. Yang and D. Heitz, "Kernelized Lagrangian particle tracking," Exp. Fluids (submitted) (2021).
[4] N. A. Malik, T. Dracos, and D. A. Papantoniou, "Particle tracking velocimetry in three-dimensional flows—Part II: Particle tracking," Exp. Fluids **15**, 279–294 (1993).
[5] N. T. Ouellette, H. Xu, and E. Bodenschatz, "A quantitative study of three-dimensional Lagrangian particle tracking algorithms," Exp. Fluids **40**, 301–313 (2006).
[6] Z. Dou, P. J. Ireland, A. D. Bragg, Z. Liang, L. R. Collins, and H. Meng, "Particle-pair relative velocity measurement in high-Reynolds-number homogeneous and isotropic turbulence using 4-frame particle tracking velocimetry," Exp. Fluids **59**, 1–17 (2018).
[7] A. Clark, N. MacHicoane, and A. Aliseda, "A quantitative study of track initialization of the four-frame best estimate algorithm for three-dimensional Lagrangian particle tracking," Meas. Sci. Technol. **30**, 045302 (2019).
[8] N. Machicoane, P. D. Huck, A. Clark, A. Aliseda, R. Volk, and M. Bourgoin, "Recent developments in particle tracking diagnostics for turbulence research," in *Flowing Matter* (Springer, Cham, 2019), pp. 177–209.
[9] C. Cierpka, B. Lütke, and C. J. Kähler, "Higher order multi-frame particle tracking velocimetry," Exp. Fluids **54**, 1–12 (2013).
[10] N. D. Cardwell, P. P. Vlachos, and K. A. Thole, "A multi-parametric particle-pairing algorithm for particle tracking in single and multiphase flows," Meas. Sci. Technol. **22**, 105406 (2011).
[11] Y. G. Guezennec, R. S. Brodkey, N. Trigui, and J. C. Kent, "Algorithms for fully automated three-dimensional particle tracking velocimetry," Exp. Fluids **17**, 209–219 (1994).
[12] S. Gupta and S. A. Vanapalli, "Microfluidic shear rheology and wall-slip of viscoelastic fluids using holography-based flow kinematics," Phys. Fluids **32**, 012006 (2020).
[13] G. Haller, "Lagrangian coherent structures," Annu. Rev. Fluid Mech. **47**, 137–162 (2015).
[14] S. Balasuriya, R. Kalampattel, and N. T. Ouellette, "Hyperbolic neighbourhoods as organizers of finite-time exponential stretching," J. Fluid Mech. **807**, 509–545 (2016).
[15] See http://cfdforpiv.dlr.de/ for "3rd workshop and 1st challenge on data assimilation & CFD processing for PIV and Lagrangian particle tracking" (2020).
[16] S. Tan, A. Salibindla, A. U. M. Masuk, and R. Ni, "Introducing OpenLPT: New method of removing ghost particles and high-concentration particle shadow tracking," Exp. Fluids **61**, 1–16 (2020).
[17] S. Rafati and N. T. Clemens, "Frameworks for investigation of nonlinear dynamics: Experimental study of the turbulent jet," Phys. Fluids **32**, 085112 (2020).
[18] X. Ma, Z. Tang, and N. Jiang, "Eulerian and Lagrangian analysis of coherent structures in separated shear flow by time-resolved particle image velocimetry," Phys. Fluids **32**, 065101 (2020).
[19] S. Krishna, M. A. Green, and K. Mulleners, "Flowfield and force evolution for a symmetric hovering flat-plate wing," AIAA J. **56**, 1360–1371 (2018).
[20] S. Qin, H. Liu, and Y. Xiang, "On the formation modes in vortex interaction for multiple co-axial co-rotating vortex rings," Phys. Fluids **30**, 011901 (2018).
[21] A. Hadjighasem, D. Karrasch, H. Teramoto, and G. Haller, "Spectral-clustering approach to Lagrangian vortex detection," Phys. Rev. E **93**, 063107 (2016).
[22] R. M. Samelson, "Lagrangian motion, coherent structures, and lines of persistent material strain," Annu. Rev. Mar. Sci. **5**, 137–163 (2013).
[23] S. C. Shadden, F. Lekien, and J. E. Marsden, "Definition and properties of Lagrangian coherent structures from finite-time Lyapunov exponents in two-dimensional aperiodic flows," Physica D **212**, 271–304 (2005).
[24] T. K. Alligood, D. T. Sauer, and A. J. Yorke, *CHAOS: An Introduction to Dynamical Systems* (Springer, 1996), pp. 193–228.
[25] S. G. Raben, S. D. Ross, and P. P. Vlachos, "Computation of finite-time Lyapunov exponents from time-resolved particle image velocimetry data," Exp. Fluids **55**, 1–14 (2014).
[26] D. Lipinski and K. Mohseni, "A ridge tracking algorithm and error estimate for efficient computation of Lagrangian coherent structures," Chaos **20**, 017504 (2010).
[27] B. Lecordier and J. Westerweel, "The EUROPIV Synthetic Image Generator (S.I.G.)," in *Particle Image Velocimetry: Recent Improvements* (Springer, 2004), pp. 145–161.
[28] M. Patel, S. E. Leggett, A. K. Landauer, I. Y. Wong, and C. Franck, "Rapid, topology-based particle tracking for high-resolution measurements of large complex 3D motion fields," Sci. Rep. **8**, 5581 (2018).
[29] C. He, Y. Liu, and L. Gan, "Instantaneous pressure determination from unsteady velocity fields using adjoint-based sequential data assimilation," Phys. Fluids **32**, 035101 (2020).
[30] P. Bartholomew, G. Deskos, R. A. Frantz, F. N. Schuch, E. Lamballais, and S. Laizet, "Xcompact3D: An open-source framework for solving turbulence problems on a Cartesian mesh," SoftwareX **12**, 100550 (2020).

Published under an exclusive license by AIP Publishing

[31]S. Laizet and E. Lamballais, "High-order compact schemes for incompressible flows: A simple and efficient method with quasi-spectral accuracy," J. Comput. Phys. **228**, 5989–6015 (2009).
[32]S. Laizet and N. Li, "Incompact3d: A powerful tool to tackle turbulence problems with up to O(105) computational cores," Int. J. Numer. Methods Fluids **67**, 1735–1757 (2011).
[33]P. Chandramouli, D. Heitz, S. Laizet, and E. Mémin, "Coarse large-eddy simulations in a transitional wake flow with flow models under location uncertainty," Comput. Fluids **168**, 170–189 (2018).
[34]E. v Sebille, S. M. Griffies, R. Abernathey, T. P. Adams, P. Berloff, A. Biastoch, B. Blanke, E. P. Chassignet, Y. Cheng, C. J. Cotter, E. Deleersnijder, K. Döös, H. F. Drake, S. Drijfhout, S. F. Gary, A. W. Heemink, J. Kjellsson, I. M. Koszalka, M. Lange, C. Lique, G. A. MacGilchrist, R. Marsh, C. G. Mayorga Adame, R. McAdam, F. Nencioli, C. B. Paris, M. D. Piggott, J. A. Polton, S. Rühs, S. H. A. M. Shah, M. D. Thomas, J. Wang, P. J. Wolfram, L. Zanna, and J. D. Zika, "Lagrangian ocean analysis: Fundamentals and practices," Ocean Modell. **121**, 49–75 (2018).
[35]A. R. Khojasteh, S. Laizet, D. Heitz, and Y. Yang, "Lagrangian and Eulerian dataset of the wake downstream of a smooth cylinder at a Reynolds number equal to 3900," Data Brief (submitted) (2021).
[36]D. Michaelis, C. Poelma, F. Scarano, J. Westerweel, and B. Wieneke, "A 3D time-resolved cylinder wake survey by tomographic PIV," in 12th International Symposium on Flow Visualisation (2006), pp. 1–11, see https://scholar.google.com/scholar?hl=en&as_sdt=0%2C5&q=A+3D+time-resolved+cylinder+wake+survey+by+tomographic+PIV&btnG=.
[37]G. E. Elsinga, F. Scarano, B. Wieneke, and B. W. Van Oudheusden, "Tomographic particle image velocimetry," Exp. Fluids **41**, 933–947 (2006).
[38]N. J. Neeteson, S. Bhattacharya, D. E. Rival, D. Michaelis, D. Schanz, and A. Schröder, "Pressure-field extraction from Lagrangian flow measurements: First experiences with 4D-PTV data," Exp. Fluids **57**, 102 (2016).
[39]Z. Sun and C. Brücker, "Investigation of the vortex ring transition using scanning Tomo-PIV," Exp. Fluids **58**, 36 (2017).
[40]F. Huhn, D. Schanz, P. Manovski, S. Gesemann, and A. Schröder, "Time-resolved large-scale volumetric pressure fields of an impinging jet from dense Lagrangian particle tracking," Exp. Fluids **59**, 81 (2018).
[41]J. F. G. Schneiders, G. C. A. Caridi, A. Sciacchitano, and F. Scarano, "Large-scale volumetric pressure from tomographic PTV with HFSB tracers," Exp. Fluids **57**, 164 (2016).
[42]F. Scarano, S. Ghaemi, G. C. A. Caridi, J. Bosbach, U. Dierksheide, and A. Sciacchitano, "On the use of helium-filled soap bubbles for large-scale tomographic PIV in wind tunnel experiments," Exp. Fluids **56**, 42 (2015).
[43]C. He and Y. Liu, "Proper orthogonal decomposition-based spatial refinement of TR-PIV realizations using high-resolution non-TR-PIV measurements," Exp. Fluids **58**, 86 (2017).
[44]A. Schröder, C. Willert, D. Schanz, R. Geisler, T. Jahn, Q. Gallas, and B. Leclaire, "The flow around a surface mounted cube: A characterization by time-resolved PIV, 3D Shake-The-Box and LBM simulation," Exp. Fluids **61**, 189 (2020).
[45]T. W. Fahringer, K. P. Lynch, and B. S. Thurow, "Volumetric particle image velocimetry with a single plenoptic camera," Meas. Sci. Technol. **26**, 115201 (2015).
[46]M. Miozzi, A. Capone, C. Klein, and F. D. Felice, "Integrating high-frequency, time resolved TSP surface maps and wake flow Lagrangian description to investigate sub critical regime in a crossflow cylinder," in 11th International Symposium on Particle Image Velocimetry (2015), pp. 1–6, see https://elib.dlr.de/99140/.
[47]R. Hain, C. J. Kähler, and D. Michaelis, "Tomographic and time resolved PIV measurements on a finite cylinder mounted on a flat plate," Exp. Fluids **45**, 715–724 (2008).
[48]D. Violato and F. Scarano, "Three-dimensional evolution of flow structures in transitional circular and chevron jets," Phys. Fluids **23**, 124104 (2011).
[49]A. Booysen, P. Das, and S. Ghaemi, "Shake-the-Box PTV in the Wake of an Ahmed Body Using Helium Filled Soap Bubbles," in 13th International Symposium on Particle Image Velocimetry (2019), pp. 301–310, see https://athene-forschung.unibw.de/128737?query=ghaemi&show_id=128895.
[50]A. R. Khojasteh, D. Heitz, Y. Yang, and S. Laizet, "Lagrangian coherent track initialisation," in 3rd Workshop and 1st Challenge on Data Assimilation & CFD Processing for PIV and Lagrangian Particle Tracking (2020).
[51]D. Schanz, M. Novara, and A. Schröder, "Shake-The-Box particle tracking with variable time-steps in flows with high velocity range (VT-STB)," in 3rd Workshop and 1st Challenge on Data Assimilation & CFD Processing for PIV and Lagrangian Particle Tracking (2020).
[52]M. Filippi, I. I. Rypina, A. Hadjighasem, and T. Peacock, "An optimized-parameter spectral clustering approach to coherent structure detection in geophysical flows," Fluids **6**, 39–34 (2021).
[53]F. A. C. Martins, A. Sciacchitano, and D. E. Rival, "Detection of vortical structures in sparse Lagrangian data using coherent-structure colouring," Exp. Fluids **62**, 69 (2021).
[54]A. R. Khojasteh, S. Laizet, D. Heitz, and Y. Yang (2021). "Lagrangian and Eulerian dataset of flow over a circular cylinder at Reynolds number 3900," Data INRAE.

Published under an exclusive license by AIP Publishing
